# Positional Cloning of “Lisch-like”, a Candidate Modifier of Susceptibility to Type 2 Diabetes in Mice

**DOI:** 10.1371/journal.pgen.1000137

**Published:** 2008-07-25

**Authors:** Marija Dokmanovic-Chouinard, Wendy K. Chung, Jean-Claude Chevre, Elizabeth Watson, Jason Yonan, Beebe Wiegand, Yana Bromberg, Nao Wakae, Chris V. Wright, John Overton, Sujoy Ghosh, Ganesh M. Sathe, Carina E. Ammala, Kathleen K. Brown, Rokuro Ito, Charles LeDuc, Keely Solomon, Stuart G. Fischer, Rudolph L. Leibel

**Affiliations:** 1 Naomi Berrie Diabetes Center, Columbia University, New York, New York, United States of America; 2 Vanderbilt University, Nashville, Tennessee, United States of America; 3 Clinical Pharmacology and Discovery Medicine, GlaxoSmithKline, Research Triangle Park, North Carolina, United States of America; 4 Discovery Technology Group, GlaxoSmithKline Pharmaceuticals, Collegeville, Pennsylvania, United States of America; 5 Center of Excellence for Drug Discovery, GlaxoSmithKline, Research Triangle Park, North Carolina, United States of America; Stanford University School of Medicine, United States of America

## Abstract

In 404 *Lep^ob/ob^* F2 progeny of a C57BL/6J (B6) x DBA/2J (DBA) intercross, we mapped a DBA-related quantitative trait locus (QTL) to distal Chr1 at 169.6 Mb, centered about D1Mit110, for diabetes-related phenotypes that included blood glucose, HbA1c, and pancreatic islet histology. The interval was refined to 1.8 Mb in a series of B6.DBA congenic/subcongenic lines also segregating for *Lep^ob^*. The phenotypes of B6.DBA congenic mice include reduced β-cell replication rates accompanied by reduced β-cell mass, reduced insulin/glucose ratio in blood, reduced glucose tolerance, and persistent mild hypoinsulinemic hyperglycemia. Nucleotide sequence and expression analysis of 14 genes in this interval identified a predicted gene that we have designated “Lisch-like” (*Ll*) as the most likely candidate. The gene spans 62.7 kb on Chr1qH2.3, encoding a 10-exon, 646–amino acid polypeptide, homologous to *Lsr* on Chr7qB1 and to *Ildr1* on Chr16qB3. The largest isoform of *Ll* is predicted to be a transmembrane molecule with an immunoglobulin-like extracellular domain and a serine/threonine-rich intracellular domain that contains a 14-3-3 binding domain. Morpholino knockdown of the zebrafish paralog of *Ll* resulted in a generalized delay in endodermal development in the gut region and dispersion of insulin-positive cells. Mice segregating for an ENU-induced null allele of *Ll* have phenotypes comparable to the B.D congenic lines. The human ortholog, *C1orf32*, is in the middle of a 30-Mb region of Chr1q23-25 that has been repeatedly associated with type 2 diabetes.

## Author Summary

Type 2 diabetes (T2D) accounts for over 90% of instances of diabetes and is a leading cause of medical morbidity and mortality. Twin studies indicate a strong polygenic contribution to susceptibility within the context of obesity. Although approximately ten genes making important contributions to individual risk have been identified, it is clear that others remain to be identified. In this study, we intercrossed obese, diabetes-resistant and diabetes-prone mouse strains to implicate a genetic interval on mouse Chr1 associated with reduced β-cell numbers and elevated blood glucose. We narrowed the region using molecular genetics and computational approaches to identify a novel gene we designated “Lisch-like” (*Ll*). The orthologous human genetic interval has been repeatedly implicated in T2D. Mice with an induced mutation that reduces *Ll* expression are impaired in both β-cell development and glucose metabolism, and reduced expression of the homologous gene in zebrafish disrupts islet development. *Ll* is expressed in organs implicated in the pathophysiology of T2D (hypothalamus, islets, liver, and skeletal muscle) and is predicted to encode a transmembrane protein that could mediate cholesterol transport and/or convey signals related to cell division. Either mechanism could mediate effects on β-cell mass that would predispose to T2D.

## Introduction

Type 2 diabetes (T2D) afflicts ∼246 million people worldwide, including ∼21 million in the United States (7% of the population); another 54 million Americans are pre-diabetic. If the incidence of T2D continues to increase at the present rate, one in three Americans, and one in two minorities born in 2000 will develop diabetes in their lifetimes [1]. Direct medical costs associated with diabetes in the United States exceed $132 billion a year [2], and consume ∼10% of health care costs in industrialized nations.

Peripheral hyporesponsiveness to insulin increases metabolic demands on the insulin-producing β-cells of the pancreatic islets. Many obese individuals are insulin-resistant, but do not become overtly diabetic provided that the increased demand for insulin is effectively met [3],[4]. However, if β-cell mass and/or function are insufficient to meet this requirement, overt hyperglycemia and T2D ensue [5]. In autopsy series of subjects with T2D, total β-cell mass is decreased [6],[7]. Primary reductions of β-cell mass predispose to diabetes in rodent models [8],[9],[10] and in autosomal dominant forms of diabetes (e.g., MODY; maturity onset diabetes of youth) [11]. Such primary reductions might predispose to some instances of T2D.

Susceptibility to T2D is strongly inherited as evidenced by the >80% concordance rates in monozygotic twins [12],[13],[14],[15], familial aggregation, and ethnic predispositions [16]. Heritability of sub-phenotypes related to T2D, e.g. insulin resistance and β-cell hypofunction is even higher [17]. Environmental factors are also important [17],[18]. Although several genes for relatively rare monogenic forms of diabetes, including MODY, syndromic (Wolfram syndrome), lipoatrophic, and mitochondrial-inherited diabetes have been identified [2],[19], the underlying genetic bases for the genetically complex T2D, accounting for >95% of diabetes patients, have remained elusive. The identification of susceptibility genes is made difficult by the polygenic nature of the phenotype [20], its reflection of convergent, distinct metabolic processes producing identical phenotypes (phenocopies), and the potent gene-gene and gene-environment (e.g. obesity) interactions that characterize the disease. Clear genetic influences on the endophenotypes (intermediate phenotypes) of β-cell mass/function and insulin resistance have been shown, and vary among racial groups. [21],[22],[23],[24]. Some notable earlier successes (e.g. *PPARG*, *CAPN10*), and a recent series of genome-wide association studies of large numbers of well-phenotyped subjects [25],[26],[27],[28],[29],[30],[31] have identified T2D susceptibility loci/genes (e.g. *TCF7L2*) whose functions with regard to the implicated phenotypes are uncertain. As no single implicated gene or allele accounts for more than a small fraction of risk to develop T2D, there are still many genes/molecular mechanisms awaiting identification.

In mice, there is striking strain-dependent susceptibility to T2D in the context of obesity [32]. We exploited the differential diabetes susceptibilities of the B6 and DBA strains segregating for the obesity mutation *Lep^ob^* [32] to identify a diabetes susceptibility QTL in B6xDBA progeny and then used congenic lines derived from the implicated interval to clone a candidate gene accounting for the QTL. Similar strategies have been used to identify QTLs (and responsible genes) for other complex phenotypes in mice [33] such as type 1 diabetes [34], diet-induced obesity [35], tuberculosis susceptibility [36], atherosclerosis [37], epilepsy [38], schizophrenia [39] and, also, T2D [40],[41],[42],[43].

We identified, “Lisch-like” (*Ll*), a novel gene, encoding multiple, tissue-specific transcripts in brain, liver and islets. The functional consequences of the hypomorphic DBA allele (diabetes-prone) in *Lep^ob/ob^* mice appear to be late embryonic to early postnatal reductions in β-cell mass due to diminished rates of β-cell replication, some “catch-up” of β-cell mass by 2–3 months, followed by mild glucose intolerance at >6 months of age. These phenotypes are recapitulated in mice with an ENU-induced null allele of *Ll*.

## Results

### Genetic Map of Diabetes QTL and Related Congenic Lines

**Figure 1:**
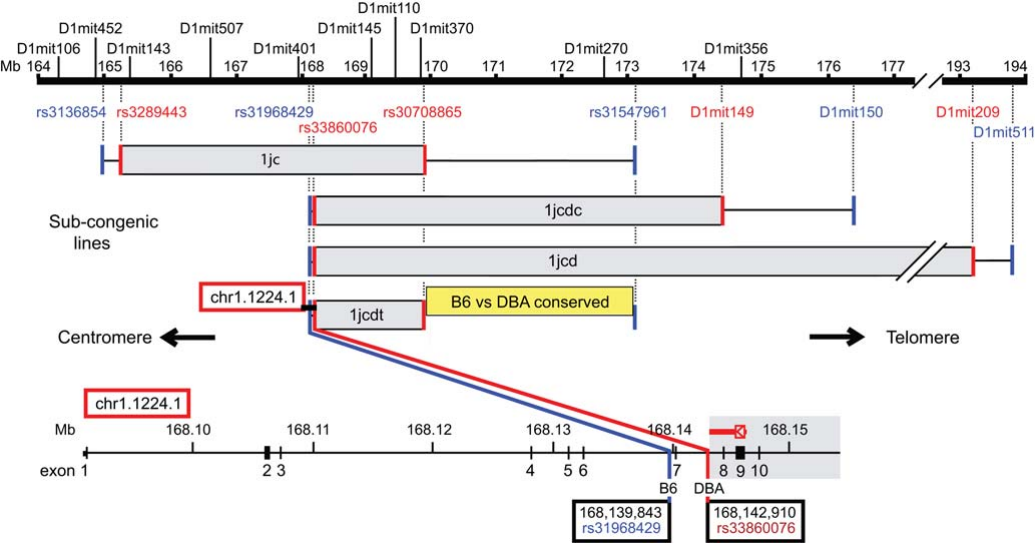
Genetic Map of Sub-Congenic Lines for Diabetes-Related Phenotypes in the Interval Chr 1 164–194 Mb. Genetic map shows sub-congenic lines (1jc, 1jcdc, 1jcd, 1jcdt) in the interval Chr1:164–194 Mb that display hypoinsulinemic hyperglycemia in association with histological evidence of a relative reduction in β-cell mass in the first 21–28 days of life due to reduced β-cell proliferation. An expanded view of the *Ll* gene (chr1.1224.1) is shown at bottom. Above the map scale, in black type, are microsatellite markers that were used to genotype B6 and DBA alleles to establish general boundaries of these congenic intervals. D1mit110 is the peak of the F2/F3 QTL linkage map (see Mapping T2D-related Phenotypes in B6xDBA F2/F3 Progeny). Below map scale, RefSNP (rs) and D-markers in red type identify DBA sequence limits of the respective congenic lines. Markers in blue type identify the closest, confirmed non-DBA (B6) sequence. Sequences in intervals between markers in red and blue type are DBA vs. B6 invariant. Gray bars are DBA-derived sequences. Yellow box corresponds to a 3.2 Mb interval, conserved between DBA and B6. The red box identifies the N-scan predicted gene, chr1.1224.1, subsequently identified as *Lisch-like* (*Ll*), extending centromerically from line 1jcdt. In the expanded view of *Ll*, the B6 boundary (rs31968429) for lines 1jcdc, 1jcd, 1jcdt is 333 bp centromeric of exon 7; the DBA boundary, (rs33860076) is 2,700 bp telomeric of exon 7. 5330438I03Rik is an anti-sense transcript described in detail in the text. Marker positions are from the mouse genome annotation (NCBI Build 36, February 2006).

We identified a QTL for diabetes-related phenotypes in obese F2 and F3 progeny of an intercross between diabetes-resistant C57BL/6J (B6) and diabetes-susceptible DBA/2J (DBA) mice segregating for *Lep^ob^*. Phenotypes including fasting blood glucose, HbA1c and islet histology mapped with LOD >8 around D1Mit110 on distal Chr 1 at 169.6 Mb (details in Methods: Mapping T2D-related Phenotypes). By producing congenic and sub-congenic B6.DBA lines also segregating for *Lep^ob^*, we refined the interval to 5.0 Mb between rs31968429 at 168.1 Mb and rs31547961 at 173.1 Mb where all four congenic lines overlap for DBA (Figure 1; details in Methods: B6.DBA Congenic Lines: Creation and Fine Mapping).

We further restricted the search (Figure 1) by identifying a haplotype block [44] conserved between B6 and DBA that extends 3.2 Mb from rs30708865 at 169.9 Mb to rs31547961 at 173.1 Mb. Only eleven unvalidated B6 vs. DBA single nucleotide polymorphisms (SNPs) in this interval are listed in the Mouse SNP database (www.ncbi.nlm.nih.gov/SNP/MouseSNP.cgi); however, among fragments we could amplify containing nine of these putative SNPs, we detected no sequence variants. Moreover, we found no coding sequence/expression difference between B6 and DBA among all genes and transcripts in the “conserved” interval by computation, direct sequencing, and quantitative mRNA expression analysis. Thus, it is unlikely that the variant(s) in the genetically-defined interval with peak at 169.6 Mb mediating differential diabetes susceptibility between these two strains is within the “conserved region.” We sequenced the 3 kb interval between rs31968429 and rs33860076 at the centromeric end of subcongenic line 1jcdt and detected no variants between the two strains. Therefore, we focused our efforts on the 1.8 Mb B6 vs. DBA “variable” interval, between rs33860076 at 168.1 Mb and rs30708865 at 169.9 Mb.

### Metabolic and Anatomic Phenotypes of Congenic Lines

The congenic/sub-congenic lines shown in Figure 1 displayed phenotypes of hypoinsulinemic hyperglycemia in association with relative reductions in β-cell mass due to reduced β-cell proliferation (see Islet Morphology and β-cell Replication and Apoptosis). Phenotypes were generally more salient in male animals. Genotype in the congenic interval (B6 or DBA) *per se* did not affect their body weight or composition. Supporting experiments are described below.

**Figure 2:**
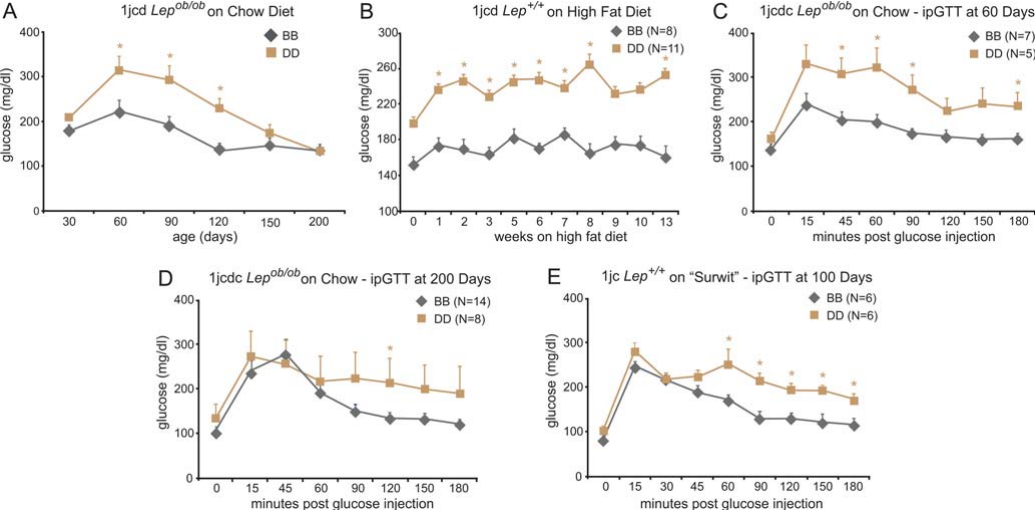
Fasting Blood Glucose and Glucose Tolerance Tests in Congenic Lines. A) Blood glucose in *Lep^ob/ob^* males congenic for the interval 1jcd (BB; homozygous for B6; DD; homozygous for DBA in the interval) and fed regular mouse chow diet (9% fat) *ad libitum*. Determinations made following a 4 h morning fast. 4–13 animals per genotype group. B) Blood glucose in *Lep^+/+^* males congenic for the interval 1jcd and fed high fat diet (60% of calories as fat) *ad libitum* for 13 wks, starting at 7 wks of age. Determinations made following a 4 h morning fast. C) ipGTT in 60-day old *Lep^ob/ob^* males congenic for the interval 1jcdc. D) ipGTT in 200-day old *Lep^ob/ob^* males congenic for the interval 1jcdc. E) ipGTT in 14-wk old male *Lep^+/+^* males congenic for the interval 1jc who had been fed the “Surwit” diet for 10 wks. In all panels, * indicates p<0.05 (2 tailed t-test for genotype effect of congenic interval; mean is +/−SEM.

By 4 weeks of age, fasting plasma glucose was elevated in *Lep^ob/ob^* males who were D/D (DBA/DBA) for the congenic interval 1jcd and fed standard (9% fat) chow; glucose concentrations were higher up to 120 days. After 120 days, there were no significant differences in fasting glucose between D/D (DBA/DBA) and B/B (B6/B6) mice (Figure 2A). The decline in pre-prandial blood glucose levels in *Lep^ob/ob^* males between 90 and 200 days is probably attributable to a slight expansion of β-cell mass in response to transient insulin resistance occurring as a normal consequence of sexual maturation (∼60 days of age) [9],[45]. To examine diabetes susceptibility in D/D animals that were obese independent of leptin deficiency, we fed lean (*Lep^+/+^*) 1jcd males a high-fat diet (60% kcal from fat) for 13 weeks, starting at 7 weeks of age. These mice became more hyperglycemic than B/B mice (Figure 2B), showing a persistence of this difference – similar to the animals in 2A – up to age ∼140 days when the study ended.

To delineate differences in acute glucose handling in D/D vs. B/B animals, we used intraperitoneal glucose tolerance testing (ipGTT). At 60 days (Figure 2C), and even up to 200 days, when the study ended (Figure 2D), *Lep^ob/ob^* 1jcdc males were less glucose tolerant than B/B. The relative reduction in glucose tolerance in D/D vs. B/B animals that are not overtly diabetic is likely related to reduction in the number of islets. The occurrence of the diabetes-related phenotype is independent of *Lep^ob^*, since 100-day old *Lep^+/+^* 1jc D/D males fed the Surwit (high fat, high sucrose) diet for 10 weeks were also less glucose tolerant than littermate B/B males (Figure 2E).

**Figure 3:**
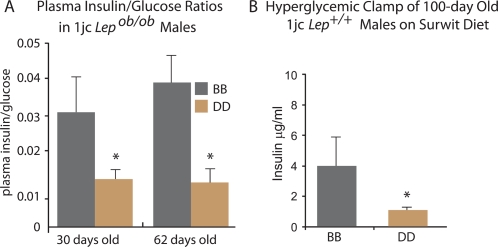
Plasma Insulin/Glucose Ratios and Hyperglycemic Clamps in Age-Grouped 1jc Congenic Males. A) Fasting plasma insulin/glucose ratios in 30- and 62-day old 1jc *Lep^ob/ob^* B/B and D/D male mice, chow-fed since weaning. Raw data are shown in Table S1. Asterisk (*) indicates significant difference between B/B and D/D animals; p-value <0.05 for 2-tailed t-test. B) Hyperglycemic clamping in 100-day old 1jc males on Surwit Diet for 18 weeks. 1jc DD male mice fed a Surwit diet for 18 wks were clamped at a blood glucose concentration of 250 mg/dl for 1 hr and serum insulin concentrations measured at 1 hr. Asterisk (*) indicates p-value <0.05 for 2-tailed t-test.

Hyperglycemia due to relative hypoinsulinemia, was evident in 1jc *Lep^ob/ob^* D/D animals fed a chow diet as early as 4 weeks (Figure 3A). At mean ages of 30- and 62-days, age-adjusted plasma insulin concentrations per mg blood glucose were lower in D/D than in B/B animals. This difference was due to lower plasma insulin in D/D (p = 0.0004) and not higher blood glucose in D/D (p = 0.916). Consistent with these ratios, D/D *Lep^+/+^* males showed a 40% decrease in insulin secretion when clamped at a blood glucose level of 250 mg/dl for an hour (Figure 3B). No difference in insulin sensitivity was detected by euglycemic – hyperinsulinemic clamping (data not shown).

**Figure 4:**
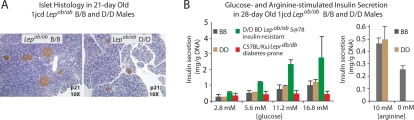
Relationship between Islet Histology and Insulin Secretion. A) Islet histology in 21-day old 1jcd *Lep^ob/ob^* B/B and D/D male mice. 4 µm pancreatic sections from 21-day old 1jcd *Lep^ob/ob^* B/B and D/D male mice were insulin-stained with anti-guinea pig IgG and visualized by light microscopy at 10× magnification. In D/D animals, islets were smaller and less numerous. By histomorphometry, the proportion of small islets (250–2000 µm^2^) in 21-day old *Lep^ob/ob^* males was greater in D/D (1jc and 1jcd) mice (73%) than in B/B (60%); whereas the proportion of large islets (10,000–50,000 µm^2^) was lower (9% in D/D and 14% in B/B). B) *In vitro* glucose-stimulated insulin secretion in pancreatic islets in 28-day old 1jc *Lep^ob/ob^* B/B and D/D males. Each congenic genotype group consisted of 3 male animals. Negative control consisted of 3 4-week old diabetes-prone *Lepr^db/db^* KsJ male animals that are hypo-responsive to glucose stimulation [9]; positive control was 3 4-week old insulin-resistant animals segregating for a diabetes-susceptibility QTL on Chr5 at 78cM, characterized by hyperglycemia and hyperinsulinemia. B/B and D/D show dose response, but no B/B vs. D/D difference at any concentration of glucose. Response to 10 mM arginine in the same animals confirms that the β-cells of the B/B and D/D congenics are comparable with regard to insulin release to a non-glucose stimulus. The 0 mM arginine control in B/B is shown to establish baseline insulin levels.

Consistent with their hypoinsulinemic hyperglycemia, 21-day old 1jcd D/D males had smaller islets than their B/B counterparts (Figure 4A). A qualitative cell-autonomous β-cell defect in insulin secretion, however, is unlikely to be the primary functional defect in D/D animals, since islets isolated from 28-day old 1jcd D/D males responded to graded glucose concentrations (2.8 mM–16.8 mM) or 10 mM arginine by secreting amounts of insulin comparable to age- and sex-matched B/B littermates (Figure 4B). Also consistent with insulin/glucose ratios and hyperglycemic clamp results, isolated islets from 60-day old 1jc *Lep^ob/ob^* males fed normal chow and 100-day old 1jc *Lep* +/+ on the Surwit diet showed reduced insulin secretion at 2.8 mM and 5.6 mM [glucose] in D/D vs. B/B littermates. For reasons indicated below, the early glucose intolerance of D/D mice is probably due, in part, to a deficiency of β-cell mass.

### Islet Morphology and β-cell Replication and Apoptosis

**Figure 5:**
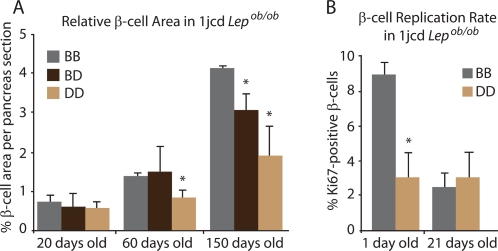
β-Cell Mass and Replication Rates in 1jcd *Lep^ob/ob^* Males. A) Relative β-cell area in 20-, 60, and 150-day old *Lep^ob/ob^* males segregating for B and D 1jcd congenic intervals. In 60 and 150-day old males segregating for the D/D sub-congenic interval, relative β-cell mass was approximately half that of B/B littermates; B/D animals were intermediate at 150 days. N = 10 for each of the 3 groups of animals. Mean+/−SEM. The asterisk (*) indicates that p<0.05 for D/D vs. B/B at 60 days, and D/D and B/D vs B/B at 150 days. These findings are consistent with *in vivo* data (see Figure 2C) showing onset of elevated blood glucose at rest and during ipGTT by 60 days. B) β-cell replication rates (Ki67) in 1- and 21-day old *Lep^ob/ob^* B/B and D/D 1jcd males. To estimate the proportion of dividing cells, the number of Ki67-positive β-cells was normalized to the total number of insulin-positive cells. Replication of β-cells in 1-day old D/D males was ∼1/3 that of B/B littermates (p = 0.017). This difference, not present in 21-day old animals, was probably due to normally reduced β-cell replication by the time of weaning. Mean+/−SEM. The asterisk (*) indicates that p<0.05 for D/D vs. B/B in 1-day old animals.

The fractional area of the pancreas accounted for by β-cells [46] in *Lep^ob/ob^* 1jcd males was examined in 20-, 60- and 150-day old mice. By 60 days a trend to reduced β-cell area was apparent in D/D, and by 150 days of age, β-cell mass of the 1jcd D/D sub-congenics was about half that of B/B littermate controls. B/D animals had β-cell masses that were about two-thirds of B/B littermate controls (Figure 5A). These findings are consistent with *in vivo* data showing onset of elevated blood glucose (see Figure 2A) and lower circulating insulin concentrations (relative to glucose) in D/D sub-congenics at ∼60 days of age (see Figure 3A), and persistence of decreased glucose tolerance at 200 days of age. The lower relative β-cell mass in D/D animals reflects fewer numbers of β-cells, rather than smaller sized β-cells. There were no differences in pancreatic weight between D/D and B/B male animals.

To assess the basis for the difference in β-cell mass by 60 days, we measured rates of β-cell replication and apoptosis. We co-stained pancreatic sections in 1jcd congenic 1- and 21-day old *Lep^ob/ob^* male mice with antibodies to insulin and Ki67 antigen, a nuclear marker of proliferation expressed during all stages of the cell cycle except G0 [47]. To estimate the proportion of dividing β-cells, we normalized the number of Ki67 positive β-cells to the total number of insulin positive cells. Groups consisted of 4 B/B and 4 D/D 1-day old mice or 4 B/B, and 8 D/D 21-day old mice. In 1-day old D/D males, the rate of β-cell replication was ∼1/3 that of B/B littermates, whereas there was no difference in 21-day old animals due to normally reduced β-cell replication by the time of weaning (Figure 5B) [48],[49],[50].

The proportion of small islets (250–2000 µm^2^) in 21-day old *Lep^ob/ob^* males was greater in D/D (1jc and 1jcd) mice (73%) than in B/B (60%); whereas the proportion of large islets (10,000–50,000 µm^2^) was lower (9% in D/D and 14% in B/B). This finding is consistent with the β-cell replication studies in P1 mice (Figure 5B), and recently reported evidence that new β-cells are derived from replication of pre-existing β-cells [51].

In 13-day old 1jc mice, when β-cell apoptosis is active [52], we did not detect significant differences between B/B and D/D islets in β-cell apoptosis using a TUNEL assay [53] and caspase-3 staining [54] (data not shown). Thus, the lower number of β-cells in D/D mice is primarily a result of lower rates of proliferation of β-cells in the perinatal period.

### Genes in the Minimal DBA Interval Conveying Diabetes Susceptibility

To identify all genes in the minimal DBA variable interval, (see above for definition) we screened 277 genes and transcripts, computationally predicted by GenScan, TwinScan, FGeneSH, Otto, or SGP2 that map to the interval. We excluded 50 single-exon transcripts (probably pseudogenes [55]) that did not belong to a transcript cluster and were not homologous to transcripts in the syntenic human interval, and 16 ribosomal gene transcripts, unique to this interval, that could not be specifically amplified due to their genomic redundancy, and manually curated the remaining 211 predicted transcripts. We rejected 63 that did not amplify in RNA/cDNA pools from multiple organs/ages of B6 and DBA mice (see Methods: Testing for Predicted Transcripts in cDNA Pools) and, using BLASTn, clustered the remaining 148 transcripts into 14 groups. These, correspond to 11 known genes and 3 predicted genes that we validated by amplification in cDNA pools.

**Figure 6:**
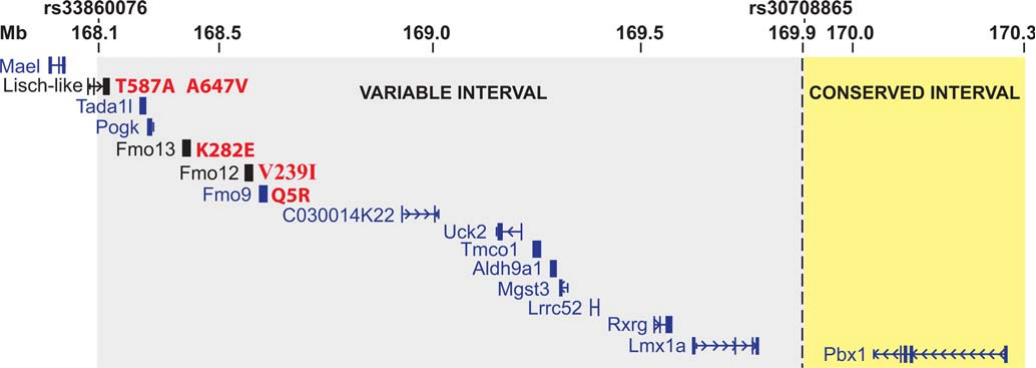
Genes in the Minimal Congenic Interval on Chr1:168.1–170.3 Mb. Gray background corresponds to the minimal DBA “variable” interval from 168.1 Mb–169.9 Mb, between markers rs33860076 and rs30708865. Yellow background corresponds to the centromeric end of the DBA vs. B6 “conserved” interval (i.e. nominally invariant). Genes in blue are from RefSeq; genes in black are predicted and locally confirmed as described in the text. The N-scan predicted gene chr1.1224.1 is designated here as “Lisch-like”. Amino acid variants are shown in red to the right of the corresponding gene. Nucleotide substitutions were confirmed by bidirectional sequencing in both C57BL/6J and DBA/2J DNA.

A map of the “variable” interval shows 14 genes, flanked by *Mael* and *Pbx1* (Figure 6). We analyzed all transcripts in the entire “variable” region.

### Analysis of Genes in the Variable Interval

The genetic variation accounting for differential diabetes-susceptibility in mice segregating B/B vs. D/D in the congenic intervals could be due to: 1) coding sequence variant(s) that alter the amino acid sequence of a protein(s); 2) regulatory variants, including anti-sense transcripts that affect expression and stability, and 3′ untranslated region (UTR) variants; or 3) splicing variants. We investigated all hypotheses.

### Non-Synonymous Sequence Variants

To identify all non-synonymous B6 vs. DBA sequence variants in the “variable” interval, we collected genomic sequence for B6 and DBA strains from databases at NCBI and Celera [56], filled gaps using bi-directional sequencing to achieve 100% coverage of all coding sequences in both strains, and validated coding sequence variants by bi-directionally re-sequencing gene fragments encompassing each variant in both B6 and DBA strains. Consequently, we identified five non-synonymous single nucleotide variants: one in each of three FMO-like (flavin mono-oxygenase) genes, and two in chr1.1224.1 (Figure 6). The latter gene, we designated “*Lisch-like*” (*Ll*) because of its sequence similarity to a gene in mouse and rat, formerly known as *Lisch*7 (http://rgd.mcw.edu/), but now known as *Lsr* (lipolysis stimulated receptor).

Computational analysis of LL and the three FMO-like proteins using SNAP [57], PolyPhen [58], SIFT [59], PAM250 matrix substitution weights [60] and PROFacc [61] predicted that all of the amino acid substitutions were benign with respect to function. The SNAP scores obtained for our variant alleles, -1 (FMO13, K282E), -2 (FMO12, V239I), -3 (LL, A647V), and -6 (LL, T587A; FMO9, Q5R), indicate that there is a ∼60%, ∼69%, ∼79%, and ∼90% respective chance of the non-synonymous variants being neutral. Similarly, PolyPhen classified all variations as “benign” and SIFT scores were well above 0.05 (neutral). PAM weights of 0 and above suggest interchangeability of the respective amino acids throughout evolution. The % differences were low, suggesting that the DBA and B6 variants are equally likely to occur in related sequences (see Methods: Computational Methods for Evaluating Effects of nsSNPs).

### Expression Differences

**Figure 7:**
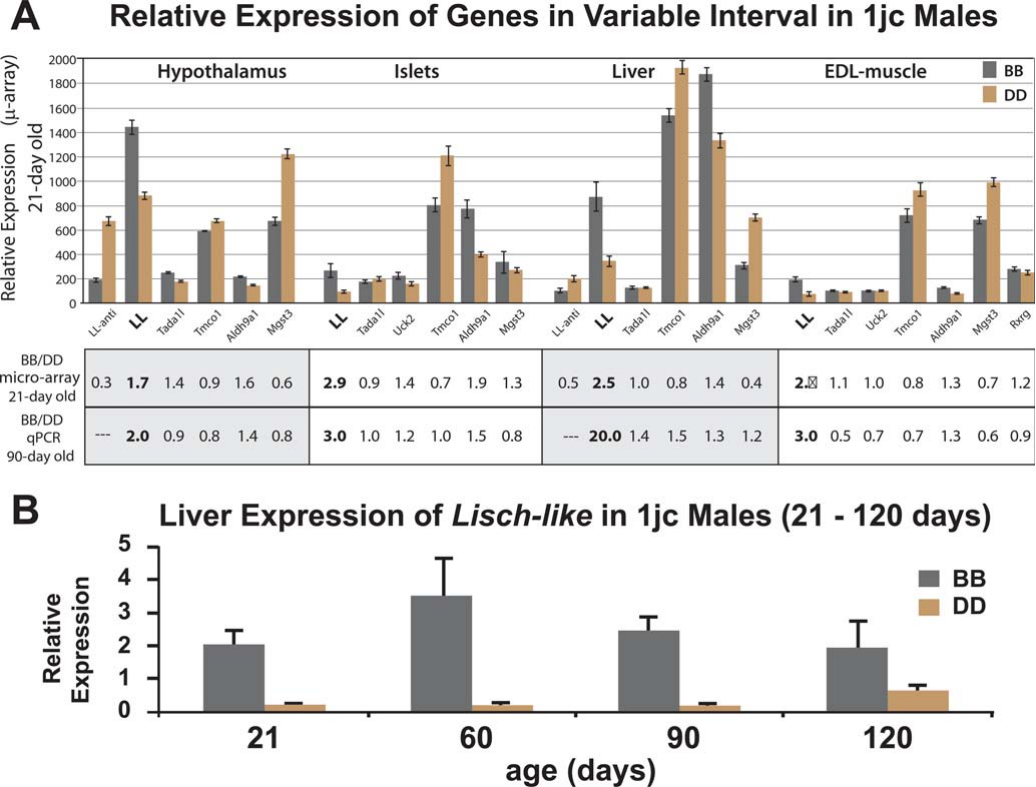
Expression Analysis of Candidate Genes and Liver Expression of *Lisch-like*. A) Tissue-specific expression analysis of genes in the “variable” portion of the minimum congenic interval. Data for relative expression (B/B to D/D) from Table 1 for hypothalamus, islets, liver and EDL-muscle are displayed graphically and numerically below the graph. 21-day old DD and BB *Lep^ob/ob^* 1jc congenic males were analyzed using Affymetrix #430A microarrays. B) Liver expression of *Lisch-like* in 1jc B/B and D/D males from 21–120 days. Samples from *Lep^ob/ob^* 1jc males were analyzed by qPCR.

We used Affymetrix microarrays to quantify those transcripts in the minimum congenic interval that we had validated by PCR-amplification (see Methods: Testing for Predicted Transcripts in cDNA Pools). We examined hypothalamus, islets, liver, soleus and EDL (extensor digitorum longus) skeletal muscle from DD and BB *Lep^ob/ob^* congenic animals (see Methods: Microarray Gene Expression Analysis). These arrays did not contain elements for all of the 14 genes we confirmed in the interval: missing from the array were the 3 FMO genes. Therefore, we also used real-time qPCR, to quantify expression of each gene and confirmed transcript in tissues and organs central to diabetes (pancreatic islets, liver, skeletal muscle, adipose tissue and hypothalamus) in 90-day old male *Lep^ob/ob^* 1jc D/D and B/B animals (see Methods: real time qPCR). Results of the microarray and qPCR experiments are shown in Table 1 and summarized in Figure 7A.

**Table 1:** BB/DD Transcript Ratios of Genes in the Variable Interval 168.1–169.9 Mb.

	BB/DD Transcript ratio									
Confirmed Genes^a^	Liver		Brain		Islets		EDL	Soleus	Muscle	Adipose
	µ-array^b^^,^ c ^,^ d ^,^ e	qPCR^f^^.^ g	µ-array	qPCR	µ-array	qPCR	µ-array	µ-array	qPCR	qPCR
*chr1.1224.1*	2.5	20^h^	1.7	2.0	2.9	3.0	2.6	4.1	2.9	2.7
*(Lisch-like)*	9×10^−4^		5×10^−7^		2×10^−3^		4×10^−4^	7×10^−4^		
***Lisch-like antisense***	0.5		0.3		NE^i^		NE	NE		
	6×10^−3^		3×10^−9^							
***Tada1l***	1	1.4	1.4	0.9	0.9	1.05	1.1	0.9	0.5	1.1
	NS^j^		1×10^−8^		NS		NS	NS		
***Pogk***	0.9	1.3	0.8	0.73	1.0	1.2	1.0	0.9	0.7	1.05
	NS		1×10^−3^		NS		NS	NS		
*FMO13*	Not on array - *inclusive-only* k ^,^ l									
*FMO12*	Not on array - *inclusive-only* k ^,^ l									
***FMO9***	Not on array - *inclusive-only* k ^,^ l									
***C030014K22***	NE	1.1	NE	1.9	NE	NE	NE	NE	1.4	1.1
***Uck2***	NE	1.4	0.6	0.8	1.4	1.2	1.0	1.3	0.7	0.8
			NS		2×10^−2^		NS	NS		
***Tmco1***	0.8	1.5	0.9	0.8	0.7	1.0	0.8	0.9	0.7	1.5
	2×10^−5^		3×10^−4^		2×10^−4^		2×10^−2^	3×10^−2^		
***Aldh9a1***	1.4	1.3	1.5	1.4	1.9	1.5	1.6	1.7	1.3	1.6
	1×10^−6^		3×10^−7^		4×10^−5^		3×10^−4^	3×10^−2^		
***Mgst3***	0.4	1.0	0.6	0.6	1.3	0.8	0.7	0.8	0.6	0.7
	1×10^−9^		2×10^−8^		NS		7×10^−6^	NS		
***Lrrc52***	NE		NE		NE		NE	NE		
***Rxrg***	NE	0.7	NE	1.6	NE	1.0	1.2	NE	0.9	1.0
							NS			
***Lmx1a***	NE	nd	NE	2.0	NE	0.9	NE	NE	1.0	1.7

a Boldface indicates RefSeq gene; regular type is locally-confirmed, predicted transcript.

b Expression profiling was carried out using Affymetrix #430A mouse gene chips.

c Ten 21-day old DD and BB *Lep^ob/ob^* 1jc congenic males were analyzed.

d Ratio is average B/B signal divided by average D/D signal in the organ.

e Lower line in µ-array box is p-value, 2-sided t-test, comparing each set of 10 BB and 10 DD mice in the same organ.

f Samples for qPCR prepared from 5 BB and 5 DD 90-day old *Lep^ob/ob^* 1jc males on 2 occasions.

g Ratios represent relative numbers of transcripts (BB/DD).

h Primer-pairs amplify transcripts of *Ll* isoforms 1, 2, 4, and 5; these comprise >90% of *Ll* transcripts.

i Expression not detected.

j Not significant (p>0.05).

k *Inclusive-only* transcripts were detected in a cDNA pool that included whole embryos, 1-day old pups, and other tissues, but not in the cDNA pool prepared from diabetes-relevant organs.

l Probes for these genes were neither on the Affymetrix #430A nor analyzed by qPCR.

Among genes in the region, including *Lmx1a* [62], and *Rxrg* [63], that constitute candidates for susceptibility to T2D, we identified no non-synonymous SNPs (nsSNPs) and no multi-organ differences in expression levels between B/B and D/D animals. The most prominent and consistent differences in expression we did observe were for chr1.1224.1 (*Ll*), which was two to four-fold lower in 21-day old *Lep^ob/ob^* D/D mice than in B/B mice in the diabetes-relevant tissues/organs by microarray analysis and up to twenty-fold lower by qPCR (Figure 7A). (We later show that *Ll* protein in hypothalamus is strikingly reduced in 1jc D/D vs. B/B; see Figure 11A). The difference in *Ll* gene expression in liver persists with age (Figure 7B) as does the difference in glucose tolerance in response to overt glucose challenge (see Figure 2D). Whether the differences in hepatic *Ll* expression are mechanistically related to differences in glucose homeostasis are unknown at this point; LL may influence hepatic gluconeogenesis, or the hepatic differences could simply mirror parallel and more physiological relevant changes in β-cells.

**Figure 8:**
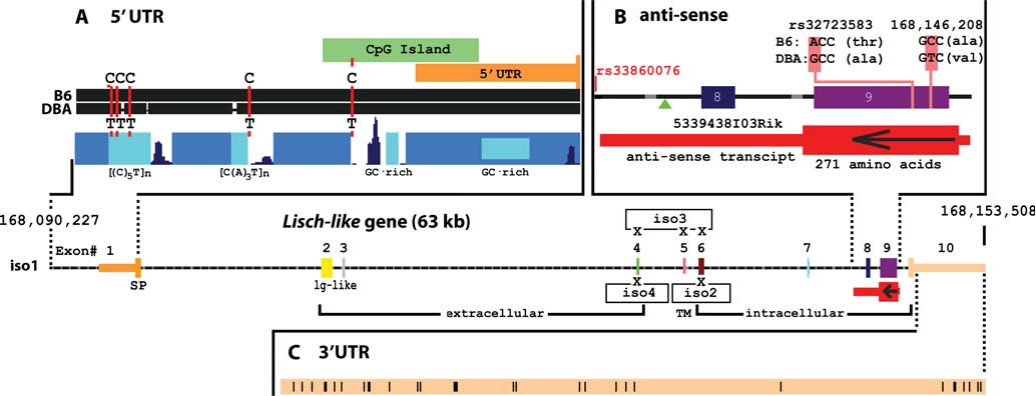
Predicted Structure of *Ll* Gene with Expanded Views of Critical Regions. *Lisch-like* gene (middle of figure) is the full-length, 10-exon, splice variant (iso1) and includes 872 bp upstream of the transcriptional start site. Predicted domains are below exons. Exon 1 includes the 5′ UTR (narrow orange bar) and cleavable signal peptide (SP). Exons 2–4 are extra-cellular, within which exons 2–3 code for an Ig-like domain. Exon 5 includes the TMD with a very cysteine-rich cluster in the carboxyl half; exons 6–10 code for a serine- and proline-rich intracellular domain; exon 10 also includes a long 3′ UTR. The “xs” identify exons deleted in isoforms 2–4. A. 5′ upstream interval (expanded view); Black bars correspond to BLAT displays vs. the reference B6 genome. DBA variants are below the DBA bar. Annotations are composites of displays from the UCSC Genome Browser on Mouse February 2006 Assembly. Royal blue peaks correspond to sequences with predicted ESPERR regulatory potential (http://www.bx.psu.edu/projects/esperr). The darker blue blocks correspond to evolutionarily conserved sequences, from the UCSC “Conservation” track. Lighter blue blocks show positions of simple sequence motifs, with the consensus motif shown below. The CpG island track, provided by the UCSC Genome Browser, was generated using the unpublished *cpglh* program from Washington University (St. Louis) Genome Sequencing Center. B. Anti-sense interval corresponds to the sequences overlapping the Riken transcript 5339438I03Rik. The green triangle identifies a 37 nt unique sequence insertion in DBA. The two non-synonymous sequence variants in exon 9 sense transcript are shown. The SNP rs33860076 is the DBA marker at the centromeric end of congenic lines 1jcdc, 1jcd, and 1jcdt. C. 3′ UTR interval; vertical black bars represent positions of 52 B6 vs. DBA nucleotide sequence variants.

We also detected (by PCR) *Ll* transcripts in e7, e11, e15, and e17 whole mouse embryos, and in testis, kidney, heart, lung, uterus, eye, thymus and spleen. For the anti-sense interval between intron 9 and intron 7 (see below and Figures 1 and 8), we found higher expression levels in liver and hypothalamus of D/D v. B/B animals. This difference is consistent with a possible suppressive role for the D/D anti-sense transcript (see below). The *Aldh9a1* gene, known to be highly expressed in human embryonic brain and involved in glycolysis and fatty acid metabolism, showed qualitative changes comparable to those seen in *Ll*. The mapping experiment that identified the interval of mouse Chr1 containing statistical signals related to T2D phenotypes would be expected to enrich for regions in which several genes might contribute to the phenotypes. Although *Aldh9a1* may be such a gene, we chose to focus initially on *Ll*, since it showed the most striking quantitative differences in expression between D/D and B/B animals.

### “Lisch-like” (*Ll*) Gene Structure and Splice Variants

#### Complete Gene Sequence

To identify 3′ and 5′ UTRs flanking the isolated transcripts of Lisch-like (*Ll*), we mapped each transcript onto the UCSC Mouse Genome Browser (http://genome.ucsc.edu/) and included contiguous 5′ and 3′ ESTs. The *Ll* gene spans 62,714 bp on mouse Chr. 1, from 168,090,795–168,153,508 (Figure 8). The full-length, 10-exon transcript, isoform 1 (iso1), is 8,279 nucleotides. It comprises a 301 nt 5′ non-coding sequence, a 1941 nt coding sequence (including stop codon), encoding a 646 amino acid polypeptide, and a 6,037 nt 3′ UTR. The predicted protein includes a cleavable, signal peptide (SP; exon 1), an extra-cellular domain (ECD; exons 2–4), a trans-membrane domain (TMD; the amino-half of exon 5), and a large intra-cellular domain (ICD; from the cysteine-rich, carboxy-half of exon 5–exon 10). Exons 2 and 3 of the ECD are immunoglobulin-like (Ig-like) V-type domains. Exon 6 is proline-rich and the ICD is overall serine/threonine-rich.

#### Isoforms

We isolated complete transcripts for 7 isoforms of *Ll* by PCR amplification of cDNAs using primer-pairs flanking the first and last predicted exons (see Methods: Cloning and Sequencing of Lisch-like Isoforms). We identified 4 major isoforms shown in Figure 8 and 3 minor isoforms. Exons 5 and 6 are absent in iso5; exon 9 is absent in iso6; and exons 5–9 are absent in iso7.

#### 5′ Upstream Interval

The 5′ upstream interval shown (Figure 8A) includes 569 nt upstream of the predicted first transcribed base of the 5′ UTR. A CpG island is predicted to overlap the 5′ UTR. By sequencing this interval in DBA BAC 95f9 (MM_DBA library, Clemson University Genomics Institute; www.genome.clemson.edu/), we discovered 8 DBA vs. B6 nucleotide variants not in the public database. Of these, only one variant, (a C to T substitution within a CpG island) is outside a repeat element.

#### Anti-sense Interval

An unspliced 2,845 nt anti-sense transcript (Figure 8B) of *Ll*, from adult male mouse B6 pituitary gland (5330438I03Rik; red bar in Figure 1), starts 42 bp telomeric of exon 9, crosses exons 9 and 8, and terminates in the intron between exons 7 and 8. This transcript (see Figure 7A) is expressed 2–3 fold higher in DBA vs. B6 in hypothalamus and liver. The centromeric end of the anti-sense transcript is just 506 bp from rs33860076 at the centromeric end of the region of DBA overlap among congenic lines 1jcd, 1jcdt and 1jcdc. An open reading frame (ORF) encodes a predicted polypeptide of 271 amino acids, but with no identifiable domain, and homologous only to ORF segments in anti-sense strands of *Ll* in other species. The interval contains 45 DBA vs. B6 variants, five of which, underlying exon 9, are listed in dbSNP. One newly discovered variant in the intron preceding exon 8, is an insertion in DBA of a 37 nt unique sequence that is homologous to a sequence in an intron of the mouse otoancorin gene on chromosome 7 and to an intronic sequence of an N-scan predicted gene on chromosome 11.

#### 3′ UTR

Of 52 B/D sequence variants in the long (6 kb) 3′ UTR of the *Ll* transcript (Figure 8C), 20 were newly discovered by our “in-house” sequencing.

### Cross-Species Comparisons of *Ll* Sequence

**Figure 9:**
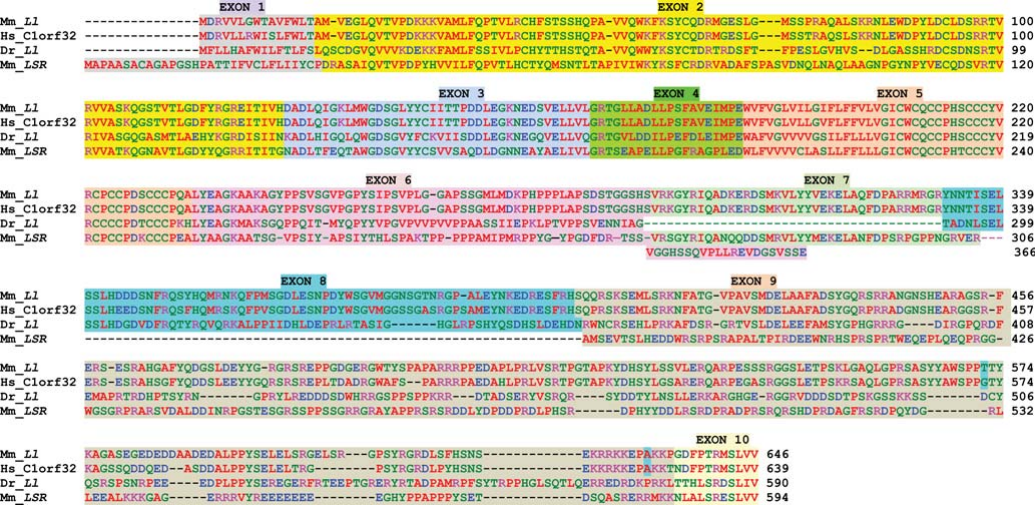
ClustalW Analysis of *Lisch-like* Homologs and the *LSR* Protein. ClustalW analysis was performed on the EMBL-EBI server (www.ebi.ac.uk/clustalw/) using their default settings. We modified the display to emphasize exonic alignments. Positions of the two non-synonymous variants in exon 9 of *Ll* are identified by blue shading. The non-homologous extension of mouse *Lsr* exon 6 (pink background) is shown beneath exon 7. Mm_Ll; *Mus musculus Lisch-like*; Hs_C1orf32, *Homo sapiens C1orf32*; Dr_Ll, *Danio rerio* (zebra fish) *Lisch-like* ortholog; Mm_LSR, *Mus musculus LSR-like* ortholog. Pair-wise similarity scores by isoform and domain are shown in Table S2.

From the Ensembl database, we identified zebra fish orthologs of *Ll and Lsr*. The clustalW pair-wise similarity scores for the predicted protein coded for by the zebra fish gene zgc:114089 (*Lsr* ortholog) is 42 vs, the mouse LSR protein, and 29 vs. the mouse LL protein. The similarity scores for the predicted protein coded for by the zebra fish gene zgc:110016 (*Lisch-like* ortholog) are 36 vs. LL and 28 vs. LSR. We performed clustalW analysis (Figure 9) between the mouse LL-iso1 protein and three related proteins: 1) the human C1orf32 protein at 1q24.1 (chr.1 165,154,620–165,211,185; NCBI Build 36.1), which is the product of a gene highly expressed in the developing human retina and brain [64]; 2) the predicted protein sequence for the zebra fish *Lisch-like* ortholog, zgc:110016 located on zebra fish chromosome 9 at 31.6 Mb; and 3) the mouse *LSR* protein, transcribed from a gene on chromosome 7 at 30.7 Mb. Pair-wise similarity scores for the intact proteins and major domains are shown in the legend. The human homolog is similar throughout, but diverges slightly in the putative ICD. The zebra fish Lisch-like ortholog and mouse LSR proteins are most alike in the TMD, less so in the Ig-like domain, and most dissimilar in the ICD. The *Lsr* protein has a short extension to exon 6, and no exon 8 equivalent. *Ll* and *Lsr* also have splicing patterns similar to the mouse *Ildr1* (Ig-like domain receptor 1) gene [65], and the proteins they encode all belong to the Lisch7 family (IPR008664; www.ebi.ac.uk/interpro).

### Knockdown of *Ll* and *Lsr* Orthologs in Zebra Fish

To assess the function of *Ll* in islet/β-cell ontogenesis, we examined expression patterns and the effects of morpholino-mediated knockdown in zebra fish embryos. Morpholinos are modified anti-sense oligonucleotides that produce a strong hypomorphic “knockdown” phenotype [66] either by inhibiting proper splicing of the pre-RNA transcript [66] or by ATG-blocking of translation [67]. Morpholino knockdown has been used to demonstrate a role for the endocrine hormones GnRH, GHRH and PACAP during development [68],[69],[70],[71]. Many of the molecular mechanisms regulating pancreas development appear to be conserved among zebra fish and other vertebrates [72], and the single zebra fish islet provides an excellent model of vertebrate development.

**Figure 10:**
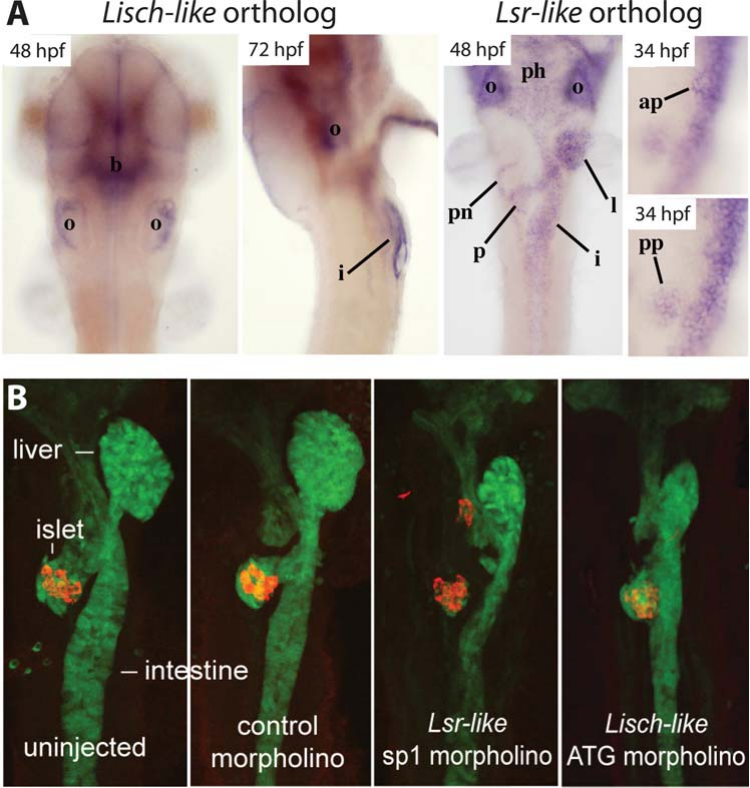
Expression Patterns and Morpholino Knockdown in Zebra Fish Embryos. A) Developmental expression of zebra fish *Lisch-like* and *Lsr-like* orthologs. *Lisch-like* RNA was hybridized *in situ* to whole-mount zebra fish embryos at 48 hours post-fertilization (hpf), dorsal view with anterior towards the top; and 72 hpf, lateral view with anterior towards the top, ventral towards the right and yolk removed. *Lsr-like* RNA was hybridized at 48 hpf and 34 hpf. *Ll* panels show ventral views of embryos with yolks removed and anterior towards the top. *Lsr-like* panels show the same image captured in the focal plane of the anterior (ap) and posterior (pp) pancreatic buds, respectively. i, intestine; ph, pharynx; pn, pronephric ducts; l, liver; ap, anterior pancreatic bud; pp, posterior pancreatic bud; p, pancreas (after anterior and posterior bud fusion); b, brain; o, otic vesicle. B) Morpholino knockdown of *Lisch-like* and *Lsr-like* orthologs at 48 hpf. Two dimensional ventral views (anterior towards top) of confocal stacks of 48 hpf embryos, uninjected or injected with 15 ng morpholino: control, *Lsr-like* sp1, and *Lisch-like* ATG. Gut-GFP transgene expression (green); insulin immunolabelling (red).

Using whole mount *in situ* hybridization (Figure 10A), we observed that the *Lisch-like* ortholog zgc:110016 was expressed in the brain and otocyst by 48 hours post fertilization (hpf), and by 72 hpf expression was evident in the intestine. The *Lsr* ortholog zgc:114089, located on Chr 15 at 39.0 Mb, was expressed in pancreas at 48 and 72 hpf, (similar to our postnatal observations in mouse with *Ll*), intestine, liver, pharynx, pronehphros and otocyst for 48 hpf (72 hpf not shown), and, at 34 hpf, in both pancreatic buds. Since the anterior bud gives rise to exocrine tissue, pancreatic duct, and a small number of endocrine cells, while the posterior bud gives rise only to endocrine tissue [69], expression of the *Lsr-like* paralog throughout this stage is consistent with a role in the ontogeny of pancreatic endocrine tissue.

The close structural similarities among Lisch-related genes (see Figure 9) suggested that functional data on both zebra fish genes could be physiologically relevant and, therefore, we studied the involvement in islet development of both orthologs. We injected (in separate experiments) morpholinos for both genes into embryos homozygous for the gut-GFP (green fluorescent protein) transgene to visualize developing endodermal organs (Figure 10B) [73]. We assessed β-cell development with an anti-insulin antibody at 48 hpf or by insulin *in situ* hybridization at 24 hpf (not shown). To assess morpholino specificity, we analyzed the effects of two separate, non-overlapping morpholinos for each gene. Both morpholinos for each ortholog independently produced similar phenotypes, providing evidence that the effects (described below) were the result of specific gene knockdown and not due to nonspecific morpholino-related effects.

Figure 10B shows that both *Lsr-like* and *Ll* morpholinos injected at 15 ng/embryo produced general developmental delay in the endodermal organs, evidenced by a smaller liver, a smaller, straighter intestine, and a smaller pancreas that does not extend as much as in wild-type. The *Lsr-like* morpholinos disrupt β-cells more severely (note ectopic insulin-positive cells in the cephalad region of the pancreas) than do the *Ll* morpholinos (note the milder local dispersion of insulin-positive cells); 48/72 and 25/144 embryos injected with morpholinos targeting *Lsr-like* and *Ll*, respectively, displayed a scattered β-cell phenotype. These effects were rarely observed in uninjected sibling embryos (0/25) or embryos injected with a control morpholino (1/35). Lower doses of *Lsr-like* and *Ll* morpholinos (∼7–10 ng) resulted in a lower frequency of β-cell scattering and higher doses (∼20–25 ng) resulted in embryonic toxicity, which is common with high doses of morpholinos. The efficacy of the splice-blocking *Lsr-like* and *Ll* morpholinos was assessed via RT-PCR and all were found to strongly and specifically inhibit proper splicing of their respective target transcripts at the 15 ng dose (not shown). In combination, the expression analyses and morpholino knockdown studies provide support for a role of *Lisch* gene family members in endodermal development, and suggest specific effects on the embryonic β-cell. The relevance of such zebra fish studies to mammalian pancreas development has been shown earlier for *Ptf1a* [74],[75] and for *Pdx1* [76].

### W87* Stop Mutation of *Ll* in C3HeB/FeJ Mice

To examine phenotypes of mice segregating for a null allele for *Ll*, we screened a repository of ENU-generated (N-ethyl-N-nitrosourea) mutant sperm DNAs from 18,000 C3HeB/FeJ G1 males (Ingenium; http://www.ingenium-pharmaceuticals.com/) for mutations in *Lisch-like* [77]. We detected a G/A substitution that encodes an amber stop mutation at threonine-87 [W87*] and also creates an EcoN1 cleavage site, which we used to genotype for the mutation. By *in vitro* fertilization, we generated W87* heterozygotes on the C3HeB/FeJ background, and bred these animals to generate progeny that were homozygous wild-type (+/+), homozygous mutant (−/−) or heterozygous (+/−) for the W87* mutation. Progeny were born at the anticipated Mendelian ratios, and the −/− animals did not appear grossly compromised.

**Figure 11:**
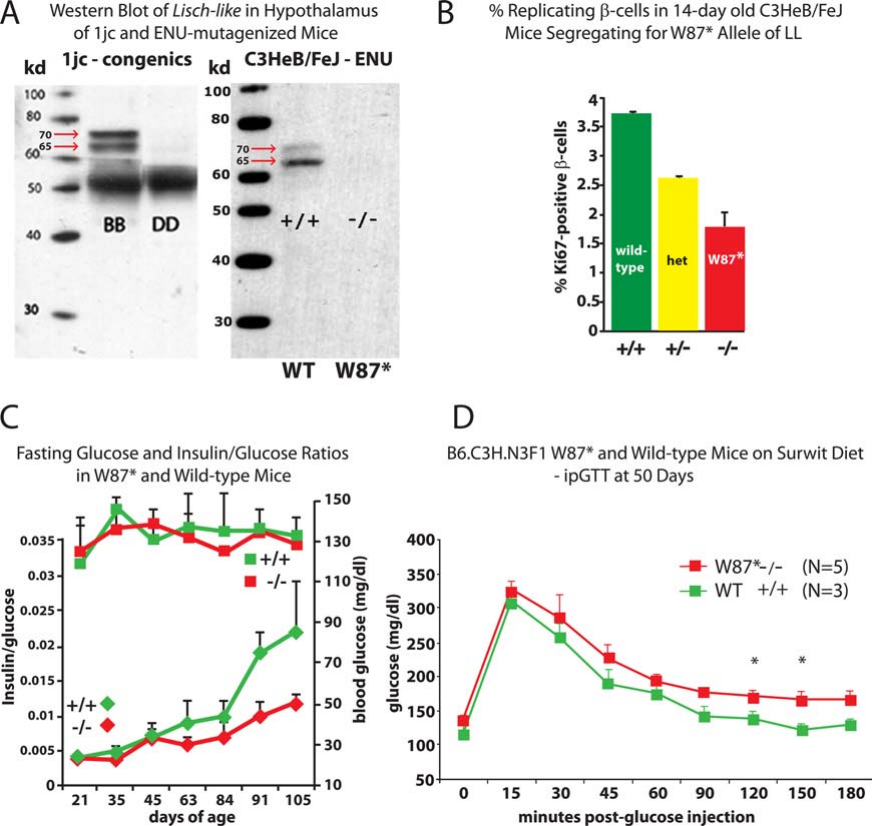
Phenotypes of Mice Segregating for the W87* Allele of *Lisch-like*. A) Western analysis of *Lisch-like* in hypothalamus of 1jc and homozygous W87* mice. The Western immunoblot shows differences in *Ll* expression in hypothalami of 1jc *Lep^ob/ob^* B/B vs. D/D congenic males (left panel), and between wild-type C3HeB/FeJ and W87* C3HeB/FeJ males (right panel). The right panel immunoblot was incubated with rabbit anti-LL antiserum, prepared against a polypeptide corresponding to exons 7 and 8 of the ICD. The antiserum had been absorbed to fixed liver extracts from knock-out mice in order to block non-specific proteins from interacting with the antibody. The LL transcript isomers are visible as a 65 and 70 kD doublet in the B/B and C3HeB/FeJ wild-type lanes, but absent in the lanes of the 1jc-D/D congenic and C3HeB/FeJ W87* homozygous ENU mutants. B) Percent Replicating β-cells in 14-day old ENU-mutagenized mice. The percentage of Ki67-positive β-cells was estimated in 14-day old C3HeB/FeJ ENU-mutagenized mice, who were either homozygous wild-type (+/+), heterozygous (+/−), or homozygous for the W87* LL amber mutation (−/−). At 14 days there was a 2-fold difference in the % of Ki67^+^ β-cells in +/+ (3.75%) vs. −/− (1.75%) ENU W87* mice; +/− were intermediate (2.5%). Non-overlapping images of longitudinal pancreatic sections (200 µm apart) were acquired and analyzed using ImageJ software version 1.37 (NIH) to count insulin-positive and Ki67^+^ cells. Pancreatic weights of +/+ and −/− were not different. C) Fasting blood glucose (squares) and insulin/glucose ratios (diamonds) in W87* (−/−) and wild-type (+/+) littermates. P-value <0.05 for 2-tailed t-test at 63 days of age. Data points at other ages show trends. D) ipGTT on 50-day old Surwit-fed B6.CH3. N3F1 W87* males. Glucose intolerance is seen in W87* mice. Mice were fasted overnight prior to dextrose injection (50% dextrose solution, 0.5 g/kg, ip). Capillary tail bleeds were performed at the specified time points to determine circulating glucose levels by glucometer (FreeStyle Flash, Abbott). Blood glucose concentrations that are marked with an asterisk are significantly different (t-test; p<0.05; mean±SEM). Area under curve +/+ vs. −/− (p = 0.02).

To verify that the W87* homozygous mutant was hypomorphic for LL protein, we compared a Western blot of hypothalamic extracts prepared from C3HeBFeJ wild-type (+/+) and mutant (−/−) mice, with a second blot of hypothalamic extracts prepared from B/B and 1jc-D/D congenic mice. We probed both sets of filters with a polyclonal rabbit antibody generated to a conjugated polypeptide, corresponding to exons 7 and 8 of isoform 1, in the predicted ICD of LL. As anticipated, LL protein was greatly reduced in the brains of D/D vs B/B congenics and in the ENU-treated W87* homozygotes vs. the wild-type animals (Figure 11A).

In mice at 14 days of age we can detect reductions in β-cell replication rates that are similar to those seen in the DD congenic lines (Figure 5B) There is a >2-fold difference in the proportion of Ki67-positive β-cells in 14-day old wild-type (3.75%) vs. homozygous W87* mice (1.75%), with heterozygotes intermediate (2.5%) (Figure 11B). Plasma insulin concentrations in *Ll* W87* homozygotes are reduced by the time of sexual maturation (Figure 11C) and, consistent with this difference, at 50 days of age, homozygous W87* males show an increased glucose AUC during iPGTT (Figure 11D). A significant decrease in β-cell mass is also detected in W87* homozygotes (1.05%±.117, n = 3, p = .0113) v. +/+ littermates (2.74±.364; n = 3) at 150 days of age.

It is important to note that these phenotypes were detected despite the segregation of the mutation on a different background strain (C3HeB/FeJ) than our congenics (C57BL/6J), and in the absence of co-segregation of the *Lep^ob^*. These preliminary data strongly support the candidacy of *Ll* as the gene accounting for the diabetes-related phenotypes of the DD congenic lines.

## Discussion

Based upon a QTL analysis of modifiers of T2D in B6xDBA F2 *Lep^ob/ob^* mice, we identified a novel gene, Lisch-like (*Ll*), whose apparent effect on β-cell development, and possibly other aspects of β-cell/islet biology, qualify it as a strong candidate mediator of susceptibility to T2D. On the C57BL/6J strain background, the presence of the DBA/2J congenic interval(s) produced mild hypoinsulinemic hyperglycemia (in association with reduced β-cell replication and mass). Our preliminary data in ENU-mutagenized mice with a null *Ll* allele are consistent with a role for LL in β-cell development.

Three of the *Ll* subcongenic lines (1jcd, 1jcdt and 1jcdc) contain only DBA DNA 3′ of exon 7, while line 1jc is DBA for the entire gene and extends DBA for another 3 Mb 5′ of *Ll*. We infer, therefore, that coding and/or non-coding DBA vs. B6 variant(s) in the region of DBA overlap accounts for the phenotypic differences between the DBA congenic lines and animals segregating for B6 alleles in this region. In the region of overlap that includes the DBA vs. B6 “variable region” (Figure 6), *Ll* is the only gene showing anticipated differences in coding sequence and gene expression. These findings strongly support, but do not prove, the putative role of *Ll* alleles in conveying the phenotypic differences seen between the various DD and BB congenic lines. The phenotypes of the *Ll* W87* C3H mice also support our inferences regarding the candidacy of *Ll* based upon the B.D congenics.

There are two non-synonymous SNPs in *Ll* within the region of overlap among the congenic lines, in exon 9. However, their effects on protein function are predicted to be minor and it is unlikely that they determine the differences in either transcript abundance or protein level seen in the congenics. Variants in other regions of the gene are likely more relevant.

In the 5′ UTR, all but one of the eight variants are in simple repeats, where they are likely less significant. The interval underlying the anti-sense transcript contains 45 D/B variants, including a long, unique insertion. A regulatory role for the *Ll* anti-sense transcript is suggested by the similar location of anti-sense transcripts at the 3′ ends of the human *C1orf32* (human ortholog of *Ll*) gene (*e.g.*, DA322725 from hippocampus), the human *LSR* gene (DA320945, also from hippocampus), the human *ILDR1* gene (AW851103), and the mouse *Lsr* gene (BY747866). Moreover, comparative inter-species transcriptomic analysis has identified the 3′ regions of transcripts as important in anti-sense regulation, and conserved overlap between species may be evidence of function [78]. For a recent review of anti-sense regulatory mechanisms, see [79].

We identified 52 B/D variants in the 3′ UTR, and it is estimated that the stability of 35% of yeast transcripts are regulated by motifs in the 3′ UTR [80]. Regulatory motifs, at a similar density, have been identified in the 3′ UTRs of several mammals, including mice [81]. A 3′ UTR polymorphism between two putative mRNA destabilizing motifs in *PPPIR3* (muscle-specific glycogen-targeting regulatory PP1 subunit) has been genetically [82] and functionally [83] related to T2D. Variants in the 3′ UTR may also affect regulation by microRNAs (miRNAs). The 3′ UTR is the target of mammalian microRNAs (miRNAs) [84] and their relevance to diabetes is underscored by the finding that mouse islet-specific miR-375 affects insulin secretion [85].

The physiological role of *Ll* is unknown. Based upon the effects of D alleles of *Ll* on β-cell proliferation rates, β-cell mass, *in vivo* insulin release and glucose tolerance, (Figure 5) it is likely that *Ll* influences early β-cell differentiation/turnover in a manner that predisposes obese animals to later failure of β-cells by effects on mass and possibly function [86],[87]. The fact that these phenotypes are substantially recapitulated in W87* *Ll* C3H mice supports this inference.

In the neonatal rodent, extensive remodeling of β-cells occurs as a result of simultaneous activation of both apoptosis and β-cell replication [49]. Between 4 and 24 weeks, postnatally, β-cell mass is estimated to increase 10 fold, related in part to increased body mass [49]. Compensation for β-cell stress/loss in adult rodents is primarily by β-cell hypertrophy and β-cell proliferation [51]. In rats, β-cell proliferation rates decline from ∼20% per day in pups, to ∼10% per day at 6–8 weeks, and to ∼2% shortly thereafter [88]. However, even this low rate of turnover apparently does not persist in adulthood. Using continuous long term BrdU labeling in B6x129Sv and BALB/C one year-old mice, replacement rates as low as ∼1/1400 mature β-cells/day have been reported [89]. Consistent with this finding, pancreas mass in the mouse was recently shown to be irreversibly constrained by the size of a progenitor pool in the embryonic pancreatic bud [87]. These data suggest that β-cell mass established in the first 6–8 weeks of life may be critical to the ability to meet subsequent stresses on β-cell function imposed by e.g. obesity, hyperglycemia, and dyslipidemia. The molecular regulation of these processes is incompletely understood, but even transient interruptions may, based upon this formulation, result in permanent effects on cell mass, or function, or both [90]. Hypoactivity of the candidate T2D modifier gene (*Ll*) reported here could mediate such effects on establishment of initial β-cell mass, and/or later responses of cell hypertrophy/replication by β-cell-autonomous effects or in response to an exogenous ligand for this putative receptor.

Observations that expression levels of *Ll* are most strikingly affected in liver, the effects of the zebra fish knockdowns on general endodermal development, and structure/function considerations raised by the homologous LSR molecule [91], are consistent with the possibility that the mechanism(s) by which *Ll* conveys effects on cell mass/function might relate, in part, to consequences of putative effects on hepatic development/ function. IGF1 [92] and hepatic growth factor [93] are examples of such β-cell “hepatokines” affecting β-cell function.

### Similarities to Trans-Membrane Receptors LSR AND ILDR1

Insight into the function(s) of the mouse *Lisch-like* protein may be gained from similarities in structure, expression, and cellular location with the human paralog, *C1orf32*, and with genes encoding related trans-membrane receptors, *Ildr1* [65] and *Lsr* [91]. Splicing patterns of these genes generate isoforms, similar to those of *Ll*. Each gene's largest isoform includes an extra-cellular Ig-like domain, a single TMD, and a similar set of ICDs in related order. In one isoform of each protein, the TMD and cysteine-rich domains are absent. An evolutionary, regulatory relationship is suggested by the observation that the *Ll*-paralog and *lldr1* are adjacent in the zebra fish genome (Zv6 assembly, UCSC Genome Browser). All three genes are abundantly expressed in the brain, liver and pancreas (and islets, where studied), and all are predicted to have 14-3-3 interacting domains (thus far experimentally verified for the human *LSR*) [94]. Although 14-3-3 interacting domains may be present on as many as 0.6% of human proteins, their occurrence on all of these Lisch-related proteins is notable, since among known 14-3-3-interacting proteins is phoshodiesterase-3B, which is implicated in diabetes and pancreatic β-cell physiology [95],[96],[97], and others, such as the Cdc25 family members, important in regulating cell proliferation and survival [98],[99].

### T2D Genetics for Region of *C1orf32*: Chr1q23

The human ortholog of *Ll*, *C1orf32*, which is 90% identical to *Ll* at the amino acid level, maps to a region of Chr1q23 that has been implicated in T2D in seven ethnically diverse populations including Caucasians (Northern Europeans in Utah) [100], Amish Family Study [101],[102], United Kingdom Warren 2 study [103], French families [104], and Framingham Offspring study [105], Pima Indians [106], and Chinese [96] with LOD scores as high as 4.3. The mouse congenic interval examined here is in the middle of, and physically ∼10× smaller than, the 30 Mb human interval. Recent analysis of the broad interval ascertained in Utah identified two peaks, one of which, at D1S2762 (at 163.6 Mb), is just 12 kb telomeric to the 5′ end of *C1orf32* [107]. The genes, and gene order, are generally conserved between mouse and human in the region syntenic to the congenic interval. The metabolic phenotypes documented in human subjects with T2D linked to 1q23 resemble diabetic phenotypes observed in congenic mice segregating for the DBA interval in B6.DBA congenics examined here [108], suggesting that the diabetes-susceptibility gene in congenic mice and human subjects may be the same gene, or among the genes, acting in the same genetic pathway(s). The syntenic interval in the Goto-Kakizaki (GK) rat also correlates with diabetes-susceptibility [109].

### Summary

We report the molecular cloning and preliminary characterization of a candidate gene for a mouse QTL modifying T2D phenotypes in mice. The gene, Lisch-like, is novel in structure among diabetes susceptibility genes, and appears to modify β-cell development. Amino acid sequence analysis is consistent with the possibility that hypomorphism for this gene could affect β-cell development by a number of possible molecular mechanisms. Proof of the role of this gene in the imputed phenotypes and molecular processes awaits its further analysis in transgenic animals and cell-based systems.

## Methods

### Animal Husbandry

Mice were housed in a barrier facility in ventilated Plexiglas cages under pathogen-free conditions at room temperature (22±1°C) with a 12 h light/dark cycle. Mice were weaned at 21 d and given *ad libitum* access to water and 9% Kcal fat Picolab Rodent Chow 20 (Purina Mills; www.purinamills.com/).The high fat diet protocol used in some animals is described below. Columbia University's Institutional Animal Care and Use Committee (IACUC) approved all protocols. After a 4 h morning fast, mice were sacrificed by carbon dioxide asphyxiation and phenotyped for weight, naso-anal length, and glycosuria. Blood was collected by cardiac puncture and aliquoted into microfuge tubes containing an anticoagulant cocktail of 10 µl of 1 mM EDTA and 1.5 mg/ml aprotinin (Sigma A-6279). Plasma and red blood cell pellets were used to measure glucose, insulin, and glycosylated hemoglobin as previously described [110]. Tissues (skeletal muscle, pancreas/pancreatic islets, liver, brain, hypothalamus, kidney, spleen, heart, visceral fat, retroperitoneal fat) were collected and immediately frozen in liquid N_2_, and stored at −80°C for further studies. Pancreata were dissected under stereoscope, weighed, and fixed in Z-fix zinc-formalin fixative (Anatech; www.anatechltdusa.com/).

### Genotyping

Liver tissue or tail tips were used for genomic DNA isolation according to standard procedures [111]. A mutation-specific assay was used to confirm that all phenotypically obese animals were *Lep^ob^/Lep^ob^* and all lean animals either +/+ or heterozygous at the *Lep* locus [112] Animals were genotyped using MapPairs Microstaellite Markers (Invitrogen; www.invitrogen.com/) as previously described [113].

### Mapping T2D-related Phenotypes in B6xDBA F2 Progeny

**Figure 12:**
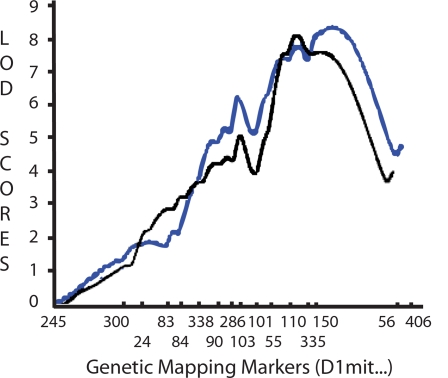
LOD Scores for Markers along Chromosome 1. LOD scores are shown for fasting blood glucose (black) and pancreatic grade (blue). Terminal phenotypes by genotype at D1Mit110 at 169.6 Mb are summarized in Table S5.

Maps were created using MapMarkerQTL (www.broad.mit.edu/genome_software/other/qtl.html) on a dataset representing 404 obese F2 progeny of a B6xDBA cross segregating for *Lep^ob^* at 120–150 days of age. The QTL for T2D was most significantly associated with fasting blood glucose, glycosylated hemoglobin, and islet histology in male mice to a region of Chr1, with peak statistical significance at D1Mit110 at 169.6 Mb from the centromere (p<10^−8^) (Figure 12). Other QTLs were identified on other chromosomes (for example Chr5 at 78cM), but none had as great an effect on the phenotype or demonstrated consistent effects on all aspects of the phenotype. We tested for interactions for QTLs and identified a modest interaction between the locus on Chr1 and a second locus at D4Mit286 (p = 0.008).

### B6.DBA Congenic Lines: Creation and Fine Mapping

B6.DBA congenic mice were generated by intercrossing *Lep^ob^/Lep^+^* B6 X DBA mice from Jackson Laboratory (www.jax.org/) to generate F1 progeny, followed by backcrossing to the recurrent B6 strain using a “speed congenic” approach in subsequent generations [114]. At the eighth backcross, a genome scan was performed in all breeders using polymorphic markers at 20 cM intervals. In the mouse line that was continued, all non-contiguous markers outside the DBA interval were homozygous B6. Over the next two generations, there were two recombination events, one that eliminated a telomeric portion of the DBA interval (line 1jc) and one that preserved approximately half of the originally defined DBA interval (line 1jcd). The 1jcd mouse was bred repeatedly to B6 mice, giving rise, by meiotic recombination, to two additional subcongenic lines (1jcdt and 1jcdc) (see Figure 1). Preservation of the phenotypes present in the original B6xDBA and DBAxB6 F2/F3 progeny was assessed by longitudinal and end-point measurements of fasting glucose, insulin, glycosylated hemoglobin and islet morphology. At N12, *Lep^ob/+^* mice B6/DBA (B/D) for the respective congenic intervals were intercrossed to produce N12F1 progeny. Obese progeny were used for fine mapping and phenotyping experiments. *Lep^ob/+^* animals D/D for the congenic interval were recurrently intercrossed or crossed to B6 *Lep^ob/+^* animals to generate *ob/ob Lep^ob^*/*Lep^ob^* animals with D/D and B/D genotypes for the Chr1 interval, respectively.

### Studies of Glucose Homeostasis

For longitudinal phenotyping studies, mice were fasted for 4 h and restrained for blood collection by a trained individual. Blood was collected from unanesthetized animals by capillary tail bleed into heparinized tubes and stored at −80°C. Glucose was measured with a FreeStyle Flash Blood Glucose Monitor (Abbott; www.abbottdiabetescare.com/). Insulin was measured by ultra-sensitive rat insulin ELISA (ALPCO; www.alpco.com/). HbA1c was measured by affinity chromatography (Mega Diagnostics; www.mega-dx.com/). Urine ketones were measured using Chemistrip Test Strips (Roche Diagnostics; http://us.labsystems.roche.com/index.shtml). For ipGTT, mice were fasted overnight and 0.5 g/kg body weight of 50% dextrose was administered intra-peritoneally at time 0. Plasma glucose was measured at 15–30 min intervals for 3 h, as above. Terminal phenotypic characterization consisted of measurements of fasting glucose, insulin, glycosuria, and glycosylated hemoglobin as previously described [110]. To control for stress-induced hyperglycemia at the time of sacrifice, tail blood glucose was also measured by glucometer one day prior to sacrifice.

### High Fat and “Surwit” Diet Studies

High fat chow pellets (#D12492i: 60% kcal from fat, 20% kcal from protein, 20% kcal from carbohydrate) and “Surwit” [115] (#D12331i; 58% kcal from fat, 16.4% kcal from protein, 25.5% kcal from carbohydrate) (Research Diets; www.researchdiets.com/) were used as described in the text.

### Morphometric and β-cell replication analysis of Pancreatic Islets

Pancreatic tissues were dissected under stereoscope to avoid contamination with adipose tissue, and weighed.

### Islet Morphometry

Non-overlapping images of longitudinal pancreatic sections were acquired and analyzed using ImageProPlus software version 5.0 (Media Cybernetics; www.mediacy.com/) to calculate insulin-positive area, insulin-positive area as % total area, and number of islets (defined by an area containing a minimum of 8 contiguous insulin-positive cells). For β-cell replication studies, we recorded the number of Ki67-positive or negative, insulin-positive cells. Replication of β-cells was expressed as % of cells (Ki67-positive and insulin-positive)/ total insulin-positive. For replication studies, ∼100 islets were examined per animal from several different non-overlapping sections through the pancreas. ImageProPlus or Image J (1.37 V; NIH) were used to determine the relative area of each section occupied by β-cells or the actual of number of β-cells for each representative longitudinal pancreatic section (50 µm apart) that had been immunochemically stained for insulin as previously described [116]. We analyzed 5–7 sections from different regions of the pancreas. Apoptosis rates were assessed using the DeadEnd Fluormetric TUNEL System G3250 (Promega; www.promega.com/) TUNEL assay and cleaved Caspase-3 (Asp175) Antibody 9661S (Cell Signaling Technology; www.cellsignal.com/).

### Pancreatic Islet Isolation

Pancreatic perfusion and islet collection were performed as previously described [117]. Each pancreas was perfused via the bile duct with 1.5 mg/ml collagenase P (Roche Applied Science; www.roche-applied-science.com/) and incubated at 37°C for 17 min. Following disaggregation of pancreatic tissue, pancreata were rinsed with M199 medium containing 10% NCS. Islets were collected by density- gradient centrifugation in Histopaque (Sigma-Aldrich; www.sigmaaldrich.com/) [117], and washed several times with M199 medium. For glucose-stimulated insulin release studies [118],[119], islets were incubated overnight in RPMI medium 1640 (Invitrogen).

### Glucose-Stimulated Insulin Secretion (GSIS)

The GSIS procedure has been described previously [120]. Islets were hand-picked into tissue culture dishes containing cold Kreb's buffer (118.5 mM NaCl, 2.54 mM CaCl_2_, 1.19 mM KH_2_PO_4_, 1.19 mM MgSO_4_, 10 mM HEPES, pH 7.4), and 2% BSA (Sigma-Aldrich), 5.5 mM glucose, and incubated overnight at 37°C. Islets were hand-picked and incubated another 15 min. in Kreb's buffer+BSA, containing 11.2 mM glucose. Hand-picked islets are then resuspended in Kreb's buffer plus BSA, supplemented with 2.8 mM glucose, and shaken at 37°C for 15 min. The pellet was spun down gently and resuspended in triplicate (5–10 islets each) in 500 µl Kreb's buffer, supplemented with glucose at final concentrations of 2.8 mM, 5.6 mM, 11.2 mM or 16.8 mM, or supplemented with 10 mM arginine and incubated for 1 h in a water bath at 37°C with constant shaking (300 rpm). After 1 h incubation, islets were gently pelleted and the supernatant collected and assayed for insulin by ELISA. Islet pellets were dissolved in high salt buffer (2.15 M NaCl, 0.01 M NaH_2_PO_4_, 0.04 M Na_2_HPO_4_, EDTA 0.672 g/L, pH 7.4) and sonicated at 4–5 W for 30 s and DNA concentration was measured using a TKO100 fluorometer (Hoefer; www.hoeferinc.com/) with Hoechst #33258 dye (Polysciences; www.polysciences.com). Results were expressed as concentration of secreted insulin/[DNA]/h.

### Testing for Predicted Transcripts in cDNA Pools

Putative transcripts, identified from public annotation and local sequencing, were validated by PCR-amplification from tissue-specific cDNA pools prepared from male and female B6 mice. Two cDNA pools were used: 1. An inclusive cDNA pool was prepared from E7 and E20 fetuses and P1 pups, and included the following tissues of 60-day old mice: eyes, large intestine, skin, tongue, spinal cord, kidney, testes/ovaries, pancreatic islets, whole brain, hypothalamus, skeletal muscle, and liver. This pool was used for transcript validation. 2. A diabetes-relevant cDNA pool, from 90-day old mice, was comprised of only the following tissues and organs: pancreatic islets, whole brain, hypothalamus, skeletal muscle, liver, and adipose tissue. This pool was used to quantify transcripts identified by computational approaches and the microarrays. Nominal intron-spanning primers were generated using the Primer3 program (www.genome.wi.mit.edu/cgi-bin/primer/primer3_www.cgi). Amplification was first performed on the diabetes-relevant pool at an annealing temperature of 60°C. If we detected no PCR-product, we performed gradient temperature PCR on the same pool using eight different annealing temperatures from 58–68°C. Gradient temperature PCR was then used to amplify the inclusive cDNA pool. If no product was detected in this pool, a 2nd set of intron-spanning primers was used before we interpreted negative amplification as failure to substantiate a predicted transcript. Positive amplification products of predicted sizes, and those that did not match the expected sizes, were gel-purified and sequenced for confirmation. The final set of primer-pairs is listed in Real-time qPCR.

### Microarray Gene Expression Analysis

RNA extraction, purification, labeling, hybridization and analysis were performed as described [121]. 10 BB and 10 DD 21-day old *Lep^ob/ob^* 1jc males were dissected and RNA was extracted from hypothalamus, liver, isolated islets, EDL muscle, and soleus muscle. Individually labeled RNA (by mouse and organ) was interrogated with Affymetrix MOE-430A expression arrays. For further details, see legends to Table 1 and Figure 7. For all transcripts in the region of interest, where possible, only probes that spanned multiple exons and clearly represented each of the 14 genes in the interval were used. If >1 probe met these conditions, we used only, the probe that gave the strongest signal. Organs were grouped into two groups by genotype and were compared using a two tailed T-test. The Affymetrix probe IDs selected for this analysis are shown in Table S3.

### Real-Time qPCR

Effects of the DBA congenic interval on the levels of confirmed transcripts expressed in diabetes-relevant organs were assessed on an organ-specific basis. We made separate pools from 90-day old *Lep^ob/ob^* 1jc D/D and B/B mice for each of the diabetes-relevant organs (see above). Each individual organ pool was generated on 2 occasions from 5 mice. RNA was extracted from organs with TRIzol acid-phenol reagent (Invitrogen). 2 µg of RNA were reverse-transcribed using SuperScript III reverse transcriptase (cDNA First Synthesis Kit, Invitrogen) with random hexamer priming. The cDNA was diluted 4-fold using nuclease-free water (QIAGEN; www.qiagen.com). 2 µl of diluted cDNA were amplified by PCR in Roche LightCycler. A standard curve for each transcript was generated using cDNA diluted 1∶1, 1∶10, and 1∶100. We assessed the number of mRNA molecules in each sample using the slope and intercepts of PCR product appearance during the exponential phase of the PCR reactions optimized for transcript-specific product using specific primers. Each sample was run in triplicate in the same LightCycler run. Using LightCycler Software, we calculated the crossing point (CP) for each sample. The CP is the first maximum of the second derivative of the fluorescence curve, and is equivalent to the number of cycles at which the fluorescence first exceeds background. In the exponential phase, the relationship between CP and initial transcript concentration is linear. We calculated relative concentration ratios, normalized to actin, as follows:
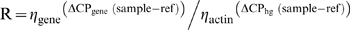



In this expression, ΔCP_gene_ is the CP of the gene in the sample minus the CP of the gene in the relevant reference; ΔCP_hg_ is the CP of the housekeeping gene in the sample minus the CP of the housekeeping gene in the reference (“ref”) sample; and η is the efficiency (where 2 is perfectly efficient) as determined by the negative slope of the plot generated when CP is plotted as a function of the log of initial concentration determined in the standard curve. Each CP listed is the mean of CP values of the triplicates for each sample. Results are summarized in Table 1. Primers used are listed in Table S4 (A).

### Cloning and Sequencing of *Lisch-like* Isoforms

We amplified full-length *Ll* cDNAs from either B6 islets (isolated by us) or from Clontech MTC Panels 1 #636745 and 3 #636757, containing pooled multiple tissue cDNAs from 8–12 week old BALB/c mice and from Swiss Webster embryos. In a final volume of 50 µl, we added 0.5 µl LA Taq (TaKaRa; www.takara-bio.com/) to a cocktail containing TaKaRa GC Buffer II, 400 µm each dNTP, 1 µl cDNA and 1 µl each primer (300 ng/µl). Primers are listed in Table S4 (B). Samples were cycled in an MJ Tetrad Thermalcycler (BioRad; www.bio-rad.com) using a Touchdown protocol of a 2 min. extension and decreasing annealing temperature from 60°C to 55°C for 10 cycles, followed by 25 cycles with an annealing temperature of 55°C. Each sample was TOPO TA cloned (Invitrogen) and plated. From all three libraries, a total of 140 colonies were picked and grown overnight in LB buffer. Inserts were amplified by colony PCR and sized by gel-fractionation. Inserts representing each unique size were then sequenced. The isoforms and the exons deleted (Δ): iso1 (intact 10 exons); iso2, Δ6; iso3, Δ4,5,6; iso4, Δ4; iso5, Δ5,6; iso6, Δ9; iso7, Δ5,6,7,8,9.

### Zebra Fish Analyses

#### A. Zebra Fish Strains and Embryo Culture

Zebra fish and embryos were raised, maintained and staged according to standard procedures [122]. The AB* (Eugene, OR) line and Tg(gut GFP)s854 transgenic line (gutGFP; [73]) were used in natural matings to obtain embryos. The gutGFP line was provided by Didier Stainier. Embryos examined at stages later than 24 hpf were maintained in embryo medium containing 0.003% phenylthiourea to inhibit pigmentation.

#### B. Morpholino Injections

Morpholino antisense and control oligonucleotides, listed in Table S4 (C), were purchased from Gene Tools (www.gene-tools.com/) and injected into 1–2 cell stage embryos at concentrations from 7–20 ng/embryo as previously described [67].

#### C. RT-PCR

Total RNA was extracted from morpholino-injected and uninjected sibling embryos at 29 hpf with TRIzol; cDNA was synthesized with SuperScript II Reverse Transcriptase (Invitrogen) using primer-pairs shown in Table S4 (D).

#### D. Immunofluorescence and RNA *in situ* Hybridization

Zebra fish gene sequences were amplified using the primer-pairs shown in Table S4 (E) and cloned into the PSTBlue-1 vector (Novagen) and used for antisense probe synthesis with T7 RNA polymerase after *XhoI* linearization (*Lsr-like*) and SP6 polymerase following *Bam*H1 linearization (*Lisch-like*). Whole-mount *in situ* hybridization was performed as described [123]. For immunofluorescence, embryos were fixed at room temperature (rt) in 4% paraformaldehyde for 2 h. After fixation, yolks were manually removed and embryos were permeabilized in acetone at −20°C for 7 min. Embryos were washed briefly in PBS +0.1% Triton ×100 (PBSTx) and incubated for 1 h in antibody hybridization buffer (PBSTx with 2% DMSO, 2% BSA and 2% sheep serum). Guinea pig anti-insulin antibody (Biomeda V2024) was diluted 1∶1000 in antibody hybridization buffer and incubated with embryos for 2 h at rt. Following antibody hybridization, embryos were washed extensively with PBSTx and incubated with Cy3-labelled donkey anti-guinea pig secondary antibody diluted 1∶500 in antibody hybridization buffer for 2 h at rt. Embryos were washed extensively with PBSTx and cleared in 80% glycerol/20% PBS. Images of optical sections were captured using a confocal microscope and 2-D projections were generated from optical sections using MetaMorph software.

### Computational Methods for Evaluating Effect of nsSNPs

We used five methods to compute the likelihood of a functional change due to single amino acid substitutions (see Figure 9). SNAP, PolyPhen, and SIFT predict changes in protein function due to a single amino acid substitution. SNAP [57] is a neural-network based method that considers protein features predicted from sequence (*e.g.*, residue solvent accessibility and chain flexibility). Scores from −9 to +9 are estimates of accuracy of prediction, computed using a testing set of ∼80,000 mutants. A low negative score indicates confidence in prediction of neutrality (functional change absent), whereas a high positive score indicates confidence in prediction of non-neutrality (functional change present). Accuracy was computed for neutrals using the equation below:
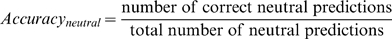



PolyPhen considers structural and functional information and alignments. Predictions are sorted into 4 classes: benign, possibly damaging, probably damaging, and unknown.

**SIFT predictions.** SIFT [59] is a statistical method that only considers alignments. Scores range from 0 to 1. Scores >0.05 indicate neutrality of a substitution.

**PAM250 matrix substitutions.** PAM matrix [124] (Percent Accepted Mutations) reflects frequency of amino acid interchange throughout evolution (by evaluating alignments of proteins in a family). Scores range from a low of −8 for rare substitutions (*e.g.* W to C) to a high of 17 (same residue found in almost all proteins in alignment).

**Percentage in alignment (PROFacc).** The score is reported as the difference in observed percentages of wild-type and mutated residues in alignments against a non-redundant UniProt [125] and PDB [126] database (at 80% sequence identity).Scores range from −100 (if the mutant is observed in all instances) to +100 (if the wild type is observed in all instances); 0 if the mutant is observed as often as the wild type. Scores near 0 favor the likelihood of a mutation being neutral.

### DBA BAC Shotgun Sequencing

BAC 95f9 DNA (5 µg) was fragmented to 1–5 kb using a nebulizer supplied with the TOPO Shotgun Subcloning kit (Invitrogen) and checked for size and quantity on an agarose gel. The shotgun library was constructed with 2 µg of sheared DNA. Blunt-end repair, dephosphorylation, ligation into PCR 4Blunt-TOPO vector, and transformation into TOP10 Electrocompetent *E. coli* were performed with the TOPO Shotgun Subcloning kit, following the manufacturer's protocol. Phenol∶chloroform extraction of the dephosphorylated DNA was replaced with Qiagen QIAquick PCR Purification spin columns (QIAGEN). Recombinant colonies were selected by blue/white screening and incubated in LB medium supplemented with 50 µg/ml ampicillin for 20 h at 37°C in 96-well deepwell plates. Plasmid miniprep was conducted in 96-well plates using QIAGEN Turbo Miniprep kits on a QIAGEN BioRobot 9600. DNA sequencing was performed on a 3730xl Genetic Analyzer (Applied Biosystems; www.appliedbiosystems.com/) using BigDye® Terminator v3.1 Cycle Sequencing Kits with M13 forward and reverse sequencing primers.

### Statistical Analyses

ANOVA and ANCOVA were used to assess effects of genotype in congenic interval. Comparisons at individual time points, or pairs of means were performed using Student's t-test. P values are 2-tailed. The Statistica package (StatSoft; www.statsoft.com/) was used for ANOVAE; Excel (Microsoft, http://office.microsoft.com/en-us/default.aspx) for t-testing.

### Western Blot

Hypothalamic extracts were prepared using M-PER Mammalian Protein Extraction Reagent (Pierce Biotechnology, www.piercenet.com/). Hypothalamic extracts (85 mg for B/B and D/D congenics and 175 mg for wild-type and mutant ENU mice) were resolved by 8% SDS-PAGE, transferred to nitrocellulose membrane (Invitrogen). We generated a set of polyclonal rabbit antibodies (Covance Research Products; www.covance.com) against the predicted ICD, spanning residues 298–401 (exons 7,8) and verified that the α-ICD rabbit antibodies detected the appropriate fusion proteins, with only minor cross-reactivity in cultured cells. We hybridized the blot with anti-LL anti-sera at a dilution of 1∶5,000 in TBS/0.05%Tween/5% milk (TBSTM) or with blocked anti-LL anti-sera diluted 1∶10,000 in TBSTM. To prepare blocked anti-sera, liver sections from C3HeB/FeJ knock-out mice were fixed overnight in phosphate-buffered paraformaldehyde at 4°C and rinsed in PBS. Sections equivalent to one-third of a liver were fragmented and mixed with 1 ml anti-sera diluted 1/1000 in PBS/0.1% Triton. Liver fragments were spun out and the supernatant was used to probe filters from ENU mice. We detected bound antibody with horseradish peroxidase-coupled antibody against rabbit IgG (Amersham Biosciences; www.amershambiosciences.com) at a dilution of 1∶5,000 using the SuperSignal West Pico Chemiluminescent Substrate kit (Pierce Biotechnology).

### Immunohistochemical and Immunofluorescnce Analysis of Pancreatic Islets

#### For β-Cell Replication Studies

Pancreata were fixed overnight in 10% formalin, embedded the specimens in paraffin, and consecutive 5 µm-thick sections were mounted on slides. For immunofluoresence and diaminobenzidine (DAB) staining of Ki67 and for insulin immunoreactivity, tissue sections were de-waxed in xylene, hydrated through a descending ethanol series and subjected to an antigen retrieval step using a heated citrate buffer solution. Several longitudinal sections >100 µm apart were used to assess β-cell replication and double staining for the nuclear proliferation marker Ki67 and insulin. Sections were incubated with Novocastra rabbit polyclonal anti-Ki67 antibody (Leica Microsystems; www.leica-microsystems.com) diluted 1∶200 and an insulin polyclonal guinea pig anti-swine antibody (Vector Lab; www.vectorlabs.com/) diluted 1∶2000 overnight at 4°C.

#### For Immunofluorescence Detection

Sections were washed in PBS and incubated with secondary anti-guinea pig IgG (1∶200) and fluorescein isothiocyanate-conjugated rabbit secondary antibody (1∶200) (Vector Labs) for 1 hr and counterstained with DAPI before the addition of mounting medium. Non-overlapping images of longitudinal pancreatic sections were acquired using a Nikon Eclipse microscope and images imported into ImageJ (1.37 V, NIH) to count insulin-positive and Ki67-insulin-positive cells. β-cell replication is expressed as % Ki67-positive+insulin-positive/total insulin-positive cells. For diaminobenzidine staining, sections were incubated with secondary biotinylated rabbit and quinea pig IgG for 1 hr and then subjected to an avidin∶biotyinylated enzyme complex (ABC Kit; Vector Labs) with DAB as substrate. Sections were counterstained with hematoxylin. Images of pancreatic sections were acquired using SpotAdvanced version 5 software (Diagnostic Instruments; www.diaginc.com/) and analyzed using Image Pro Plus software to calculate the % of β-cell area occupied by Ki67-positive cells. We examined 30–50 islets per animal from several non-overlapping sections through the pancreas.

### Accession Numbers

Genbank (www.ncbi.nlm.nih.gov/(Genbank) accession numbers for the *M. musculus* genes: *Lisch-like*, lipolysis-stimulated remnant receptor-related (XM_001473525); Lsr (NM_017405); *Ildr1* (NM_134109); *Tada1l* SPT3-associated factor 42 (NM_030245); *Pogk* pogo transposable element with KRAB domain (NM_175170); *FMO13*, flavin-containing monooxygenase family; FMO-like (XM_136366); *FMO9*, flavin-containing monooxygenase family; FMO-like (NM_172844) *FMO12*, flavin-containing monooxygenase family; FMO-like (XM_136368); *C030014K22Rik*, unknown (NM_175461); *Uck2*, uridine monophosphate kinase (NM_030724); *Tmco1*, membrane protein of unknown function (NM_001039483); *Aldh9a1*, aldehyde dehydrogenase 9, subfamily A1 (NM_019993); *Mgst3*, microsomal glutathione-S-transferase 3 (NM_025569); *Lrrc52*, leucine-rich repeat (LRR) protein of unknown function (NM_00103382); *Rxrg*, retinoid X receptor, gamma (NM_009107); *Lmx1a*, LIM homeobox transcription factor 1, α (NM_033652); *Pbx1* (NM_008783); *H.sapiens C1orf32* (NM_199351); *LSR* (NM_015925); *ILDR1* (NM_175924); *D.rerio Ll* ortholog, zgc:110016 (NM_001030192.1); *D. rerio Lsr* ortholog, zgc:114089 (NM_001025472.1); *R. rattus Lsr* (NM_032616).

The Genbank accession numbers for protein sequences: *M. musculus* Lisch-like (amino acid residues 150–795, XP_001473575); (Lsr) (NP_059101); Ildr1 (NP_598870); *H. sapiens* C1orf32 (NP_955383); LSR (NP_057009); ILDR1 (NP_787120); *D. rerio* Lisch-like (NP_001025363); *D. rerio* Lsr (NP_001020643); *R. rattus* Lsr (NP_116005)

## Supporting Information

Table S1 Data for Figure 3A: Plasma Insulin/Glucose Ratios in Age-Grouped 1jc *Lep^ob/ob^* Males.
(0.03 MB DOC)

Table S2 Pair-Wise Similarity Scores by Isoform and Domain for Figure 9: ClustalW Analysis of *Lisch-like* Homologs and the *LSR* Protein.
(0.03 MB DOC)

Table S3 ID numbers for Affymetrix MOE-430A Probes used in Methods: Microarray Gene Expression Analysis.
(0.03 MB DOC)

Table S4 Oligonucleotide Sequences.
(0.05 MB DOC)

Table S5 Data for Figure 12: Terminal Phenotypes in 404 Obese F2 Progeny by Genotype at D1mit110.
(0.04 MB DOC)
