# Supplementary material for: Positional Cloning of “Lisch-like”, a Candidate Modifier of Susceptibility to Type 2 Diabetes in Mice
Source: PLoS Genet. 2008 Jul 25;4(7):e1000137. doi: 10.1371/journal.pgen.1000137 (PMC2464733; doi:10.1371/journal.pgen.1000137)
Supplement: Table S1 — Data for Figure 3A: Plasma Insulin/Glucose Ratios in Age-Grouped 1jc Lepob/ob Males. (0.03 MB DOC) [file pgen.1000137.s001.doc]

**Table S1. Data for Figure 3A: Plasma Insulin/Glucose Ratios in Age-grouped 1jc *Lep^ob/ob^* males.**

| strain | age | # of mice | glucose (mg/dl) | insulin (ng/ml) | insulin/glucose |
| --- | --- | --- | --- | --- | --- |
| B/B | 30 days | 9 | 221.8 (40.50)^a^ | 4.94 (1.26) | 0.031 (0.010) |
| D/D | 30 days | 19 | 317.6 (37.89) | 2.95 (0.44) | 0.013 (0.003) |
| B/B | 62 days | 17 | 485.6 (34.97) | 16.29 (2.69) | 0.039 (0.007) |
| D/D | 62 days | 9 | 464.1 (42.65) | 5.32 (1.41) | 0.012 (0.004) |
| ANCOVA (p)^b^ | | | 0.916 | 0.004 | 0.007 |

**^a^**Standard deviation in parentheses

**^b^**Age-adjusted genotype effect
