# Supplementary material for: Positional Cloning of “Lisch-like”, a Candidate Modifier of Susceptibility to Type 2 Diabetes in Mice
Source: PLoS Genet. 2008 Jul 25;4(7):e1000137. doi: 10.1371/journal.pgen.1000137 (PMC2464733; doi:10.1371/journal.pgen.1000137)
Supplement: Table S2 — Pair-Wise Similarity Scores by Isoform and Domain for Figure 9: ClustalW Analysis of Lisch-like Homologs and the LSR Protein. (0.03 MB DOC) [file pgen.1000137.s002.doc]

**Table S2. Pair-wise Similarity Scores by Isoform and Domain for Figure 9: ClustalW Analysis of *Lisch-like* Homologs and the *LSR* Protein.**

| Protein | residues (#)**^a^** | Full-length**^b^** | Ig-like Domain**^c^** | TMD**^d^** | ICD**^e^** |
| --- | --- | --- | --- | --- | --- |
| *H.sapiens* C1orf32 | 639 | 90 | 98 | 98 | 87 |
| *D.rerio* *Ll*-paralog | 629 | 36 | 51 | 70 | 26 |
| *M. musculus* *Lsr* | 594 | 34 | 47 | 70 | 25 |

**^a^**Number of amino acid residues in largest isoforms of each protein.

**^b^**Pair-wise similarity scores between LL (iso1) and each of the three Lisch-related proteins.

**^c^**Pair-wise similarity scores between LL Ig-like domain and Ig-like domains of Lisch-related proteins.

**^d^**Pair-wise similarity scores between LL transmembrane domain and TMDs of Lisch-related proteins.

**^e^**Pair-wise similarity scores between LL intra-cellular domain and ICDs of Lisch-related proteins.
