# Supplementary material for: Positional Cloning of “Lisch-like”, a Candidate Modifier of Susceptibility to Type 2 Diabetes in Mice
Source: PLoS Genet. 2008 Jul 25;4(7):e1000137. doi: 10.1371/journal.pgen.1000137 (PMC2464733; doi:10.1371/journal.pgen.1000137)
Supplement: Table S3 — ID numbers for Affymetrix MOE-430A Probes used in Methods: Microarray Gene Expression Analysis. (0.03 MB DOC) [file pgen.1000137.s003.doc]

**Table S3. ID numbers for Affymetrix MOE-430A Probes used in Methods: Microarray Gene Expression Analysis.**

| **Gene** | *LL anti-sense* | *Lisch-like* | *Tada1l* | *Pogk* | *C030014K22Rik* |
| --- | --- | --- | --- | --- | --- |
| **Probe^a,b^** | 1436894_at | 1436293_x_at | 1424427_at | 1459896_at | 1440242_at |
| **Gene** | *Uck2* | *Tmco1* | *Aldh9a1* | *Mgst3* | *Lrrc52* |
| **Probe** | 1448604_at | 1423759_a_at | 1437398_a_at | 1448300_at | 1432913_at |
| **Gene** | *Rxrg* | *Lmx1a* | *Pbx1* |  |  |
| **Probe** | 1418782_at | 1421554_a | 1449542_at |  |  |

**^a^**Where possible, selected probe spanned multiple exons.

**^b^**Probes used gave strongest signal for each gene.
