# Supplementary material for: Positional Cloning of “Lisch-like”, a Candidate Modifier of Susceptibility to Type 2 Diabetes in Mice
Source: PLoS Genet. 2008 Jul 25;4(7):e1000137. doi: 10.1371/journal.pgen.1000137 (PMC2464733; doi:10.1371/journal.pgen.1000137)
Supplement: Table S4 — Oligonucleotide Sequences. (0.05 MB DOC) [file pgen.1000137.s004.doc]

**Table S4. Oligonucleotide sequences.**

**A. Primer-pairs used in Methods: Real-time qPCR.**

| **Gene** | **Forward Primer** | **Reverse Primer** |
| --- | --- | --- |
| **Actin** | AGCCATGTACGTAGCCATCC | CTCTCAGCTGTGGTGGTGAA |
| *Lisch-like* | ATCTGCTGGTGCCAATGCTG | GCCGTACGAGTCTGCGAAGG |
| ***Tada1l*** | TGGGCCAACCTGAAGTTGTGGT | GCCCTTGGGTTTTCCAGGCT |
| ***Pogk*** | CTGAATTTGACCCTGAAAGAAGAGC | ACTTCCCGGTAGAGGGC |
| *FMO13* | AGGTTTAACCATGCCAATTATGGAC | CTCTGGGGCTTTTCACAAACT |
| ***FMO9*** | TGGAGCTTGGTCGTGTAG | CCACGGTGCCATCATCAA |
| *FMO12* | AATTCTGGAGCAGATGTGGC | CATGGTCCCAAACTCGATTC |
| ***C030014K22*** | ATCGTCCTGCGCTACAAGACCC | GGGTCACAGTCTCTGTCGTGTTCC |
| ***Uck2*** | GGGAGCGTGCGTCGGT | AGGACTCGGTAGAAGCTATCCTGGC |
| ***Tmco1*** | GCAGACACGCTGCTCATCGT | CGCGAACATGGATTTCATCCGTACC |
| ***Aldh9a1*** | ACGGGAAGTCCATATTTGAGGCCC | GGAGGCGCACCCGCTTT |
| ***Mgst3*** | GGCGCACGAAGGTGAGCC | CCTCGATACCGCTTGCTAGGGT |
| *Lrrc52* | ACCGGATTGCACATCATCGACCA | CCCCGCTCGACGTTCGGA |
| ***Rxrg*** | CAGTAGCCTTGCCCACGGG | ACCTGGTAAGGGCTTGATGTCCT |
| ***Lmx1a*** | CTTCGAGGCCATTGCGCCC | GGGTCGCTTATGGTCCTTGCCG |

**B. Primer-pairs used in Methods: Cloning and Sequencing of *Lisch-like* Isoforms.**

| **Gene** | **Forward Primer** | **Reverse Primer** |
| --- | --- | --- |
| ***Ll* (full-length)** | AGCCATGTACGTAGCCATCC | CTCTCAGCTGTGGTGGTGAA |

**C. Oligonucleotides used in Methods: Zebra Fish Analyses; B. Morpholino Injections.**

| morpholino | Sequence (5’ -3’) **^a^** | locus **^b^** |
| --- | --- | --- |
| *lsr-like* sp1 | atgttgagtgtacTTGAGCTGGCTC | chr15:38,994,445-38,994,469 |
| *lsr-like* sp2 | gaatgaaacacacTTCCTCCAGCAT | chr15:38,994,596-38,994,620 |
| *Ll-*ATG | AGCGTGTAACAAAAACATGATCCAG | chr9:31,645,414-31,645,438 |
| *Ll-* splice | CAACTTTGCActgtgccaaagaaag | chr9:31,641,215-31,641,239 |

**D. Primer-pairs used in Methods: Zebra Fish Analyses; C.** RT-PCR.

| Gene | Forward primer | Reverse primer |
| --- | --- | --- |
| *Lsr-like* | TGCCTATGCAAATGGGAGTTGGTG | ttggcaacctctcgctccatgtaa |
| *Lisch-like* | GCAAACTAACCCGCACTAAACTGG | AGGGACTCAGGAAAGGTGAAGGAA |
| ef1α | CAAGGGCTCCTTCAAGTACGCCTG | GGAAGAATGGCATCAAGGGCA |

**E. Primer-pairs used in Methods: Zebra Fish Analyses; D. Immunofluorescence and**

**RNA *in situ*** hybridization.

| Gene | Forward primer | Reverse primer |
| --- | --- | --- |
| *Lsr-like* | CACGGACTTTCTCTACATACTTTTG | TTCATCCACATCATCGTACACT |
| *Lisch-like* | TTTCACTGCAAAGTTGTGATGGCG | ATGTCATCCAGCACACCTGTCC |

**^a^**Intronic sequences in lower case.

**^b^**Position is from the March 2006, Zv6 assembly.
