# Supplementary material for: Positional Cloning of “Lisch-like”, a Candidate Modifier of Susceptibility to Type 2 Diabetes in Mice
Source: PLoS Genet. 2008 Jul 25;4(7):e1000137. doi: 10.1371/journal.pgen.1000137 (PMC2464733; doi:10.1371/journal.pgen.1000137)
Supplement: Table S5 — Data for Figure 12: Terminal Phenotypes in 404 Obese F2 Progeny by Genotype at D1mit110. (0.04 MB DOC) [file pgen.1000137.s005.doc]

**Table S5. Data for Figure 12: Terminal phenotypes in 404 obese F2 progeny by genotype at D1mit110**.

| **Sex** | **Female** | | | **Male** | | |  |
| --- | --- | --- | --- | --- | --- | --- | --- |
| **Genotype** | **B/B** | **B/D** | **D/D** | **B/B** | **B/D** | **D/D** | **P-value^a^** |
| **Weight (g)** | **61.1** | **63.5** | **57.5** | **60.3** | **56.4** | **54.0** | **0.001** |
| **HbA1c (%)** | **8.7** | **9.6** | **12.2** | **11.6** | **14.1** | **14.3** | **0.00001** |
| **Plasma Glucose (mg/dl)** | **565** | **645** | **773** | **646** | **770** | **784** | **0.00001** |
| **Plasma Insulin (µU/ml)** | **258.3** | **272.2** | **178.5** | **235.1** | **83.1** | **148.1** | **0.01** |
| **Pancreatic Grade^b,c^** | **2.4** | **2.7** | **3.7** | **2.7** | **3.5** | **3.6** | **0.00001** |
| **Pancreatic Insulin (µU/mg)** | **64674** | **35963** | **7696** | **15890** | **9445** | **8284** | **0.00001** |
| **Pancreatic Insulin/Glucagon** | **0.80** | **0.54** | **0.12** | **0.25** | **0.17** | **0.13** | **0.00001** |
| **Islets (#)** | **18.0** | **17.6** | **11.5** | **13.6** | **9.8** | **10.9** | **0.005** |
| **Hyperplastic Islets (#)** | **3.0** | **2.3** | **0.9** | **1.7** | **0.5** | **0.6** | **0.0001** |
| **Average Islet Size (mm^2^)** | **0.025** | **0.021** | **0.017** | **0.022** | **0.017** | **0.017** | **0.00001** |
| **Total Islet Area (mm^2^)** | **0.53** | **0.42** | **0.22** | **0.31** | **0.17** | **0.20** | **0.0001** |
| **Islet Area/Total Area (%)** | **2.1** | **1.6** | **0.9** | **1.3** | **0.7** | **0.8** | **0.00001** |

**^a^**P-value is calculated for effect of genotype.

**^b^**Pancreatic grade is a subjective measure of number and size of islets and islet integrity.

**^c^**Grading is from 1 (many, large, intact islets) to 5 (few, small islets with little insulin staining).
